# A silent H-bond can be mutationally activated for high-affinity interaction of BMP-2 and activin type IIB receptor

**DOI:** 10.1186/1472-6807-7-6

**Published:** 2007-02-12

**Authors:** Dionys Weber, Alexander Kotzsch, Joachim Nickel, Stefan Harth, Axel Seher, Uwe Mueller, Walter Sebald, Thomas D Mueller

**Affiliations:** 1 Lehrstuhl für Physiologische Chemie II, Theodor-Boveri Institut für Biowissenschaften (Biozentrum) der Universität Würzburg, Am Hubland, D-97074 Würzburg, Germany; 2 Protein Structure Factory, c/o BESSY GmbH, Albert-Einstein-Str. 15, D-12489 Berlin, Germany; 3 Virchow Research Center, Versbacher Str. 9, D-97078 Würzburg, Germany

## Abstract

**Background:**

Bone morphogenetic proteins (BMPs) are key regulators in the embryonic development and postnatal tissue homeostasis in all animals. Loss of function or dysregulation of BMPs results in severe diseases or even lethality. Like transforming growth factors β (TGF-βs), activins, growth and differentiation factors (GDFs) and other members of the TGF-β superfamily, BMPs signal by assembling two types of serine/threonine-kinase receptor chains to form a hetero-oligomeric ligand-receptor complex. BMP ligand receptor interaction is highly promiscuous, i.e. BMPs bind more than one receptor of each subtype, and a receptor bind various ligands. The activin type II receptors are of particular interest, since they bind a large number of diverse ligands. In addition they act as high-affinity receptors for activins but are also low-affinity receptors for BMPs. ActR-II and ActR-IIB therefore represent an interesting example how affinity and specificity might be generated in a promiscuous background.

**Results:**

Here we present the high-resolution structures of the ternary complexes of wildtype and a variant BMP-2 bound to its high-affinity type I receptor BMPR-IA and its low-affinity type II receptor ActR-IIB and compare them with the known structures of binary and ternary ligand-receptor complexes of BMP-2. In contrast to activin or TGF-β3 no changes in the dimer architecture of the BMP-2 ligand occur upon complex formation. Functional analysis of the ActR-IIB binding epitope shows that hydrophobic interactions dominate in low-affinity binding of BMPs; polar interactions contribute only little to binding affinity. However, a conserved H-bond in the center of the type II ligand-receptor interface, which does not contribute to binding in the BMP-2 – ActR-IIB interaction can be mutationally activated resulting in a BMP-2 variant with high-affinity for ActR-IIB. Further mutagenesis studies were performed to elucidate the binding mechanism allowing us to construct BMP-2 variants with defined type II receptor binding properties.

**Conclusion:**

Binding specificity of BMP-2 for its three type II receptors BMPR-II, Act-RII and ActR-IIB is encoded on single amino acid level. Exchange of only one or two residues results in BMP-2 variants with a dramatically altered type II receptor specificity profile, possibly allowing construction of BMP-2 variants that address a single type II receptor. The structure-/function studies presented here revealed a new mechanism, in which the energy contribution of a conserved H-bond is modulated by surrounding intramolecular interactions to achieve a switch between low- and high-affinity binding.

## Background

Bone morphogenetic proteins (BMPs) and other members of the transforming growth factor-β (TGF-β) superfamily, like the activins, growth and differentiation factors (GDFs) and TGF-βs are secreted signaling proteins that regulate the development, maintenance and regeneration of tissues and organs [1-4]. Their importance in the development of multicellular organisms is visible from their existence in all vertebrates and non-vertebrate animals. The number of different TGF-β members correlates with the complexity of the organism, with four members found in *C. elegans *[5], seven members in *D. melanogaster *[6] and more than 30 members in men [7]. Dysregulation of signaling of TGF-β like proteins leads to a variety of diseases, including skeletal malformations [8], osteoporosis [9], cardiovascular and metabolic diseases [10], muscular disorders [11], and cancer [12].

Members of the TGF-β superfamily bind two different types of serine/threonine-kinase receptors termed type I and type II receptors [2,13,14]. Both receptor subtypes share a common architecture, i.e. a small extracellular ligand binding domain, a single transmembrane segment and a cytoplasmic serine/threonine-kinase domain. The kinase domains of type I and type II receptors share a high level of amino acid sequence similarity. However a glycine/serine-rich segment – the GS box – in the membrane-proximal part of the intracellular domain is unique to the type I receptors. In general, ligand binding induces hetero-oligomerization of type I and type II receptors initiating the intracellular signaling cascade. The constitutively active type II serine/threonine-kinase transphosphorylates the type I receptor at the GS box thereby activating the type I kinase [15]. The latter subsequently activates SMAD proteins, which dimerize and migrate to the nucleus, where they, in concert with other proteins, function as transcription factors to regulate responsive genes [16,17]. Two SMAD pathways exist. SMAD-2/-3 are activated by activins and TGF-βs and SMAD-1/-5/-8 are activated by BMPs and a subset of GDFs. Recent discoveries however show that other signaling pathways involving the MAP kinase pathway or small G proteins like Ras might be directly addressed by TGF-β members [18]. Proteomics approaches also identified various adaptor and other proteins associated with the intracellular domain of the BMP type II receptor suggesting that signaling of TGF-βs and BMPs might be more complex than the well-examined SMAD pathway [19].

Signal transduction of TGF-β proteins is highly controlled at several levels; a manifold of modulator proteins in the extracellular space vary the activities of these factors [20]. Although they are often termed antagonists there are also examples of modulator proteins leading to an increase in receptor-mediated activity [21]. Pseudo receptors as well as co-receptors can either inhibit or modulate signaling at the membrane surface level [22,23]. Inside the cell various possibilities exist to adjust or interrupt the activity, e.g. by inhibitory SMAD proteins, phosphatases to counteract the receptor kinase activity [24], ubiquitination-dependent proteolysis of the receptors/SMADs [25,26] or by transcriptional repressors [27].

One important feature of the TGF-β superfamily is the limited specificity of its ligand-receptor interactions. For more than 30 ligands only seven type I receptors and five type II receptors are known. Thus one receptor of a particular subtype has to bind several different ligands. But even though the ligands outnumber the available receptors, several BMPs and GDFs have been shown to interact with several different receptor chains of both type I and type II. However, preferences seem to exist. For instance BMP-2 uses preferentially BMPR-IA and BMPR-IB less so [28], GDF-5 prefers BMPR-IB [29] and BMP-7 ActR-I [30]. An especially intriguing situation exists with the type II activin receptors ActR-II and ActR-IIB which interact with different BMPs, activins, GDF-8/-11 and Nodal [15,31]. Ligand specific patterns seem to exist for type I and type II receptors. Recent clinical and biochemical studies on GDF-5 have shown that the receptor specificity profile of a ligand can be absolutely crucial for its biological functions [32,33]. This underlines the importance of understanding the molecular mechanisms by which these relative binding affinities are generated.

In the present study we analyze the interaction of BMP-2 with its type I receptor BMPR-IA and its type II receptor ActR-IIB. BMP-2 is a prototypical member of the TGF-β superfamily with respect to ligand-receptor promiscuity. It binds with high-affinity to both type I receptors BMPR-IA and BMPR-IB; three different type II receptors, i.e. BMPR-II, ActR-II and ActR-IIB can be recruited to yield a signaling hetero-oligomeric complex [15,34]. The usage of the activin type II receptors is especially interesting as they exhibit a dual specificity and affinity [29,30,35,36]. ActR-II and ActR-IIB bind activin A (Act-A) with high affinity in the low nanomolar range leading to activation of the SMAD-2/-3 pathway, whereas binding of BMP-2 occurs with low-affinity in the micromolar range resulting in the activation of the SMAD-1/-5/-8 pathway. Since Act-A and BMPs can exhibit opposing activities [37,38], which are regulated by competition for the activin type II receptors, it is important to know how the binding affinity to both ligand subgroups activins and BMPs can be changed by orders of magnitude.

Here we describe in a structure-/function analysis how binding specificity of the type II receptor ActR-IIB for different ligands as well as of BMP-2 for different type II receptors is encoded on a single-amino acid level. A single mutation in BMP-2 selectively enhances the binding affinity for BMPR-II. Exchange of two other amino acids results in a nearly 100-fold increase in affinity particular for type II receptor ActR-IIB without affecting the binding affinities to the other receptors. Remarkably, a "silent H-bond" is thereby converted into a hot spot of binding energy, which also exists in Act-A.

## Results

### Architecture and assembly of the ternary ligand-receptor complex

**Figure 1:**
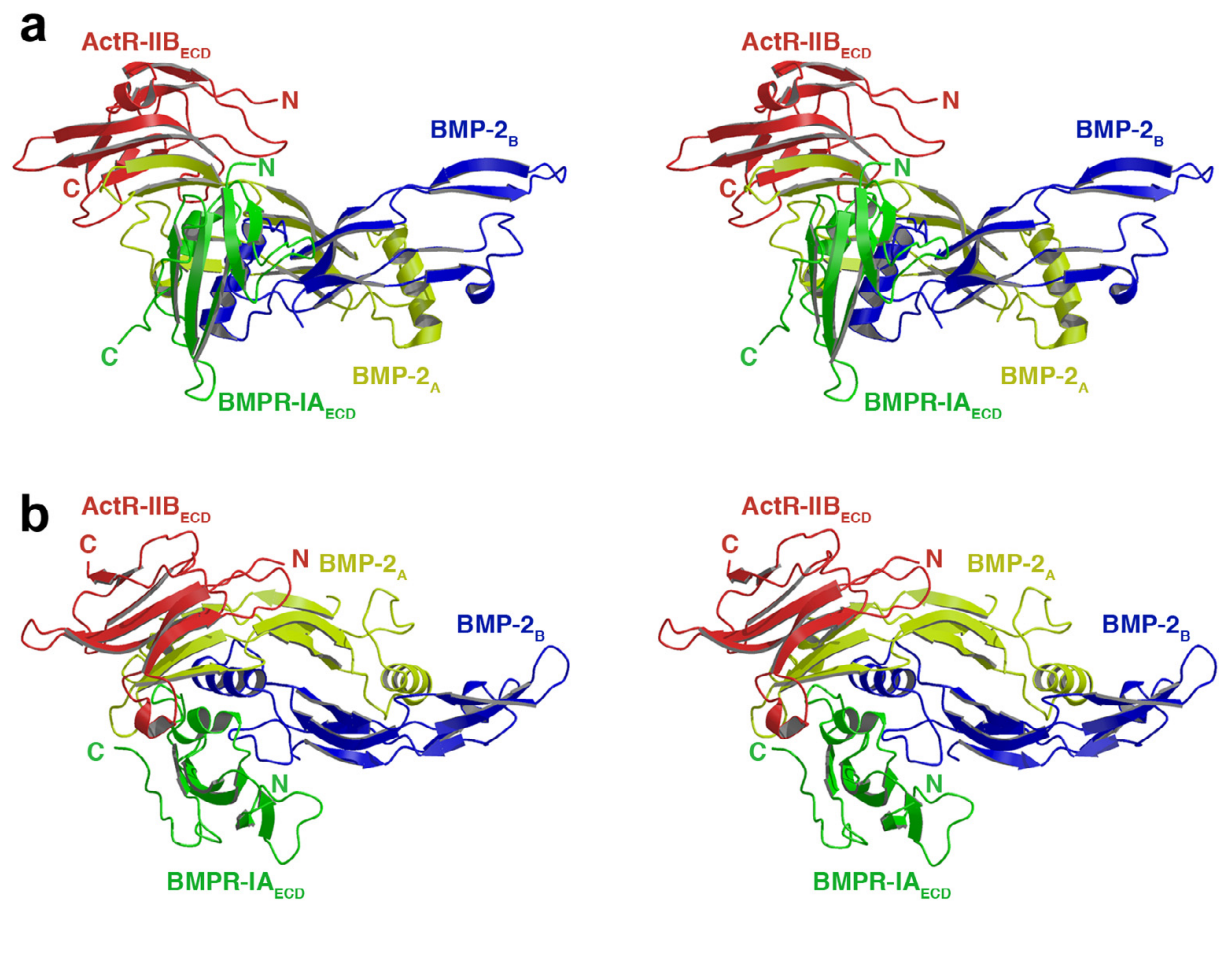
**Ternary ligand-receptor complex of wildtype BMP-2**. Ribbon representation (stereo figure) of the crystal structure of wildtype BMP-2 (monomers in yellow and blue) bound to one receptor ectodomain of BMPR-IA_ECD _(green) and ActR-IIB_ECD _(red), **(a) **viewed from the side, **(b) **or from above. The unexpected stoichiometry 1:1:1 is due to crystal packing forces resulting in the loss of one BMPR-IA_ECD _and one ActR-IIB_ECD _molecule in the ternary complex.

The crystal structure of the ternary ligand-receptor complex of wildtype BMP-2 shows a dimeric ligand bound to only one BMPR-IA_ECD _and ActR-IIB_ECD _occupying the expected wrist (type I receptor binding site) and knuckle epitopes (type II receptor binding site). Both receptor ectodomains are located on the same half of the dimeric ligand (Fig. 1), with the binding sites being almost identical to those identified in the crystal structures of the complexes BMP-2:BMPR-IA [39] and BMP-7:ActR-II [40]. The change in ligand-receptor stoichiometry in the crystal is clearly due to crystal packing as SDS-PAGE and RP-HPLC analyses proved this complex to be hexameric in solution (see Additional file 1). Inspection of the crystal lattice contacts shows that symmetry-related proteins block the empty epitopes.

**Figure 2:**
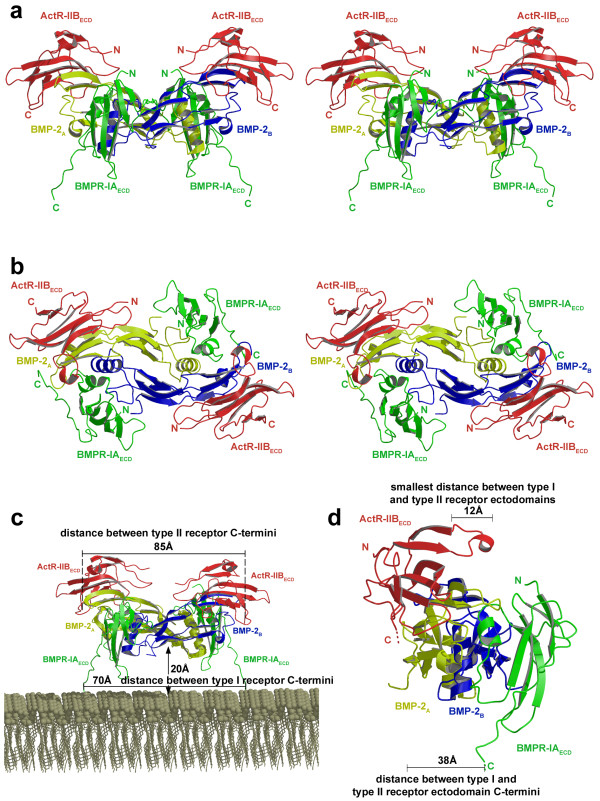
**Ternary ligand-receptor complex of BMP-2 variant L100K/N102D**. Ribbon representation (stereoview) of the ternary complex of the BMP-2 double variant L100K/N102D (in yellow and blue) bound to BMPR-IA_ECD _(green) and ActR-IIB_ECD _(red), viewed from the side **(a) **or from above **(b)**. **(c) **Distances between the C-termini of the receptor ectodomains of each subtype are indicated. **(d) **The shortest distance between BMPR-IA_ECD _and ActR-IIB_ECD _occurs between the two receptor ectodomains located on the same half of the BMP-2 dimer across the β-sheet of BMP-2 and measures ~12 Å. No direct receptor-receptor contacts between the ectodomains of either subtype as proposed for the TGF-β:TGF-β receptor interaction [44, 47, 61] can be observed.

In a second analysis a BMP-2 variant with enhanced affinity for ActR-IIB (see below) was used for complex preparation and crystallization. Crystals from this complex comprising the BMP-2 variant L100K/N102D were obtained under different crystallization conditions and exhibit distinct morphology. Analysis of this complex yielded a hexameric ligand-receptor arrangement with all receptor binding epitopes occupied as expected for the dimeric BMP-2 (Fig. 2a,b).

Inspection of the receptor-ligand interactions of both BMP-2/receptor complexes (comprising BMP-2 L100K/N102D, in the following termed ternary complex (1:2:2), or wildtype BMP-2, in the following termed ternary complex (1:1:1)) demonstrates that the ligand-receptor interfaces are highly similar. Superposition of the complexes yields an r.m.s. deviation of 0.3 Å for the Cα atoms of BMP-2 (in the ternary complex (1:1:1) only "bound" parts of BMP-2 were considered), and 0.4 Å for the Cα of BMP-2 and ActR-IIB_ECD _(see Additional file 2). Thus, the different crystal forms and the employed mutant protein did not alter the general fold or the assembly of BMP-2 and the receptors. This indicates that the core parts of these proteins are rigid and that no major conformational changes take place during complex formation.

The type I receptor BMPR-IA_ECD _occupies the wrist epitope of BMP-2 (Figs. 1 and 2a,b), whereas the type II receptor ActR-IIB_ECD _is located on top of finger 1 of BMP-2 consistent with the knuckle epitope identified by mutagenesis [28,39]. This binding site overlaps almost perfectly with that of ActR-IIB in the structures of the Act-A:(ActR-IIB_ECD_)_2 _complex [41,42]. A comparison with the ActR-II binding site in the structure of the complex BMP-7:(ActR-II_ECD_)_2 _[40] shows that the site of ActR-II however is shifted slightly away from the fingertips. In the recently published structure of another BMP-2 ternary ligand-receptor complex comprising of BMPR-IA_ECD _and ActR-II_ECD _[43], the binding site of ActR-II is, however, identical with that of ActR-IIB in our complex structures. This observation suggests that the type II receptor location in the knuckle epitope is probably dependent on the nature of the ligand, here BMP-2 or BMP-7. The slight shift of the epitope might represent one possible mechanism for generating ligand specific receptor recognition.

In contrast, in other TGF-β ligand-receptor complexes, e.g. Act-A:ActR-IIB_ECD _and TGF-β3:TβR-II, the ligand structures differ vastly in the free and bound form [41,42,44,45]. The cause for the large changes in the dimer architecture of Act-A and TGF-βs is yet unclear, but is probably not due to receptor binding itself. Inspection of the backbone dynamics in TGF-β3 using NMR-relaxation methods reveals an inherent flexibility in the TGF-β molecule [46], which might result in an dynamic equilibrium between an open and a closed dimer architecture for TGF-βs and possibly also activins [47]. In contrast, BMP-2 seems not to change its overall dimer architecture upon binding to either type I or both receptor subtypes. Accordingly, in the absence of gross conformational changes binding affinities of ActR-IIB for BMP-2 alone or for BMP-2 complexed with BMPR-IA_ECD _are identical (see Additional file 3). Our current structural data also excludes the possibility that the binding cooperativity for the type II receptor observed for BMP-2 in cell-based experiments [2] results from direct contacts between the receptor ectodomains as proposed for the TGF-βs [44,47]. The closest proximity between the ectodomains of either subtype measures about 12 Å (Fig. 2c,d). Involvement of the intracellular domains of the receptors in the generation of binding cooperativity has been ruled out by binding experiments using truncated receptors [40].

An alternative model suggests interaction of the transmembrane segments as a possible source for cooperativity. However, the distance between the C-termini of both ActR-IIB_ECD _(Fig. 2c) is about 85 Å and the C-termini of BMPR-IA_ECD _are separated by approx. 70 Å. The distance between the traceable C-termini of the receptor ectodomains BMPR-IA and ActR-IIB measures about 40 Å. Modeling of the missing C-terminal peptide sequences – no electron density is observed for the six C-terminal residues of BMPR-IA_ECD _and 20 residues of ActR-IIB_ECD _– shows that contacts between all four transmembrane helices are impossible due to steric restraints by the ligand. Only hetero-dimeric interactions between the transmembrane helices of one BMPR-IA and one ActR-IIB receptor residing on the same half of the BMP-2 ligand are possible (see also discussion in Allendorph *et al*. [43]).

### Ternary complex formation does not alter type I ligand-receptor core interface

Although the global fold of BMP-2 is not affected by the binding of both receptor subtypes, small locally restricted changes in backbone and side chain conformations are observed in the wrist and knuckle epitopes of both ternary complexes. The binding of BMP-2 to its high-affinity receptor BMPR-IA_ECD _causes a local induced fit in the so-called pre-helix segment (Pro48-Asn56) of BMP-2 [48]. In free BMP-2 this segment exhibits high temperature factors but upon type I receptor binding temperature factors within this segment drop to low values also observed in the core of the binary complex BMP-2:BMPR-IA_ECD_.

**Figure 3:**
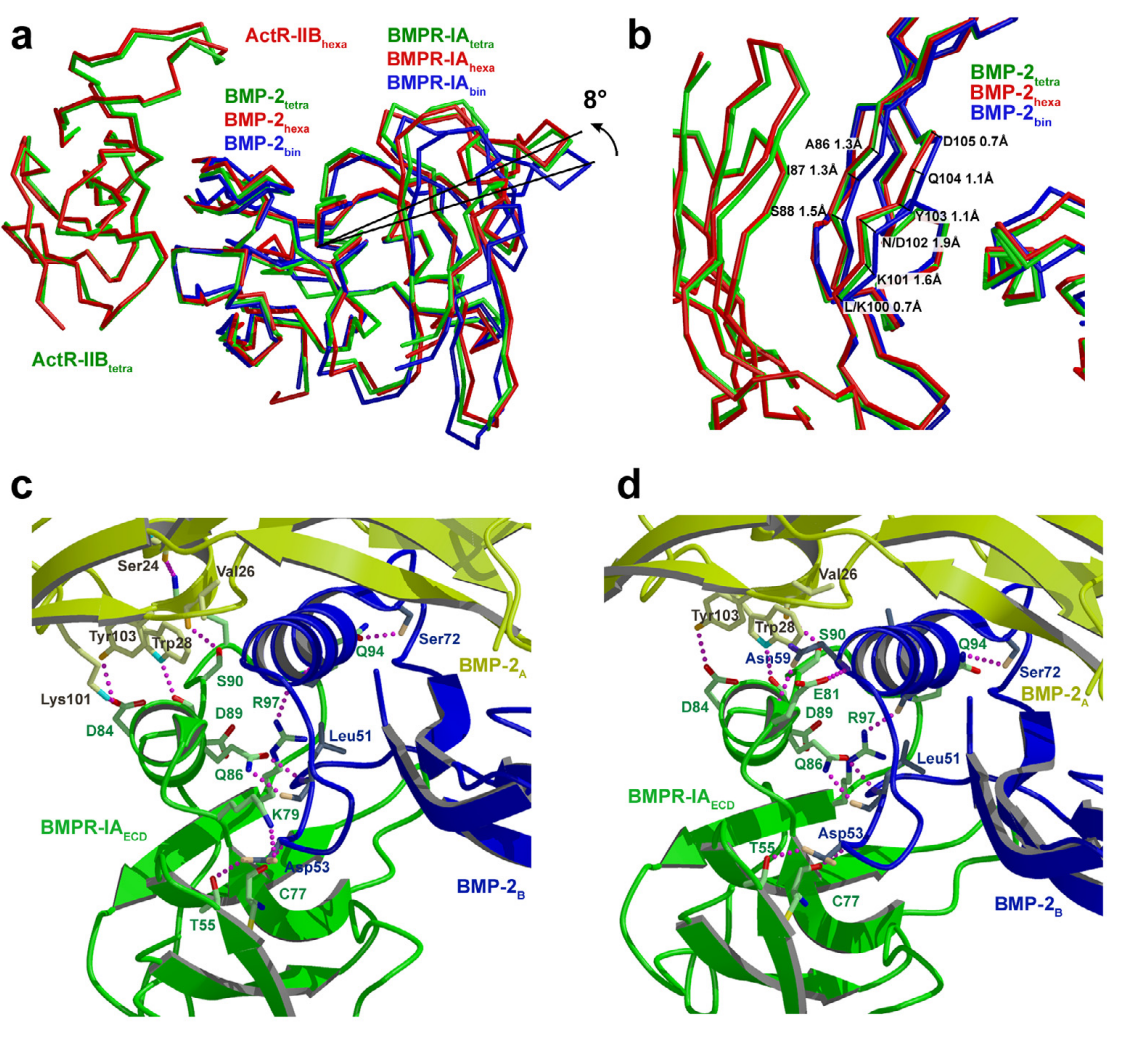
**BMP-2 type I receptor interface**. **(a) **The tilt angle of BMPR-IA bound to BMP-2 changes upon binding of the type II receptor ActR-IIB. A superposition of the structures of BMP-2:(BMPR-IA_ECD_)_2 _(blue, PDB entry 1REW), the ternary complex (1:1:1) of wildtype BMP-2:BMPR-IA_ECD_:ActR-IIB_ECD _(green) and the ternary complex (1:2:2) of BMP-2L100K/N102D:(BMPR-IA_ECD_)_2_:(ActR-IIB_ECD_)_2 _(red) is shown. The comparison of both assemblies reveals that the rearrangement is not due to the mutations introduced in BMP-2L100K/N102D. **(b) **A change in the backbone conformation of residues 86 to 88, and 100 to 105 located in the finger 2 of BMP-2 is the possible cause for the tilt angle change. The type I ligand-receptor interfaces of the ternary (1:1:1) **(c) **and (1:2:2) **(d) **BMP-2/receptor complexes are structurally almost identical differing only in very few H-bonds that are located at the solvent accessible surface.

Interestingly, binding of ActR-IIB to the binary complex BMP-2:BMPR-IA_ECD _results in a small but significant reorientation of BMPR-IA. This reorientation is identical in both ternary complexes, comprising wildtype BMP-2 as well as the double variant BMP-2L100K/N102D. Thus the allosteric change in the assembly is neither dependent on the stoichiometry nor on the difference in affinity to the type II receptor ActR-IIB. The BMPR-IA molecule changes its tilt angle by about 8° compared to the binary complex of BMPR-IA bound to BMP-2 (Fig. 3a). The axis of rotation runs through the type I interface core, i.e. residue Gln86 of BMPR-IA, perpendicular to the dyad of the complex. The movement of BMPR-IA seems to be initiated by small structural changes in the backbone conformation of residues 86 to 88 and 100 to 105 in the β-strands 6 and 7 of finger 2 of BMP-2. Both strands move towards ActR-IIB up to 1.5 Å and this change seems to be propagated to the wrist epitope (Fig. 3b). The structure of BMPR-IA remains unaltered and the BMP-2 molecule is therefore the sole source of the observed structural plasticity. As a result, residues located far from the axis of rotation show the largest displacement, e.g. residues in the loops β1β2 and β3β4 of BMPR-IA move by almost 4 Å (Fig. 3a). In contrast, residues in the type I ligand-receptor interface core are not affected; nine intermolecular hydrogen bonds (H-bonds) observed in the core of the wrist epitope of BMP-2 and BMPR-IA_ECD _of the binary complex are also found in both ternary complexes (Fig. 3c,d, see Additional file 4) and their geometrical parameters (e.g. hydrogen bond length and angles) are preserved.

For the recently published structure of the ternary ligand-receptor complex of BMP-2 bound to BMPR-IA and ActR-II, no such rearrangement for the type I receptor binding has been described [43]. A detailed comparison of this complex structure with those of this study reveals that the change in type I receptor orientation is significantly smaller; the tilt angle of BMPR-IA changes by only 3° compared to its location in the BMP-2:BMPR-IA_ECD _binary complex. Consistently, the change in backbone conformation of the β-strands 6 and 7 of BMP-2 in the complex of Allendorph *et al*. is also smaller, e.g. the distance between the Cα atoms of Asn102 of the binary and ternary complex is 1 Å (for comparison the distance between the Cα atoms of Asn102 of the binary complex and the ternary complexes of this study is roughly 2 Å, see Fig. 3b). Although the physiological role of the type I receptor rearrangement is yet unknown – our BIAcore study clearly shows that type II receptor ectodomain binding to BMP-2 is independent of the presence of a type I receptor – the comparison suggests that the different change in type I receptor orientation might be dependent on the nature of the type II receptor.

Analysis of solvent molecules in the BMP-2 type I receptor interface has shown that the main-binding determinants of the BMP-2 – type I receptor interaction are surrounded by tightly bound water molecules, which seem to play an important role in the ligand-receptor recognition mechanism. Of these water molecules in the type I receptor-ligand interface of the binary complex [48], all (four in the core, three additional in the periphery) are found in identical positions in the ternary complexes of this study and the ternary complex structure published by Allendorph et al. [43] although the crystallization conditions differ significantly. This corroborates our hypothesis that interface water molecules play an important role in the ligand-type I receptor recognition possibly by generating a major part of the structural plasticity of BMP-2 [48]. In summary, despite the reorientation of BMPR-IA upon recruitment of the type II receptor ectodomain, the binding epitope of the type I receptor remains unaltered.

### The ligand-type II receptor core interface is predominantly hydrophobic

**Figure 4:**
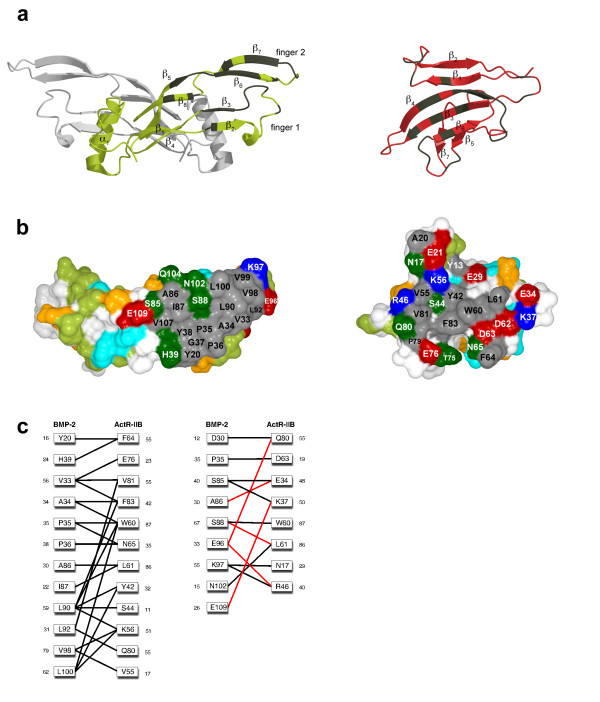
**BMP-2 type II receptor interface**. **(a) **Location of the type II ligand/receptor binding epitopes on wildtype BMP-2 (left) and ActR-IIB_ECD_(right). For designation of β-strands and finger-like structures see [62], the contact residues are marked in grey. **(b) **Surface representation of the type II ligand/receptor binding epitopes in the ''open book'' view. The surface of BMP-2 (left) is color coded by amino acid properties as follows: hydrophobic amino acids (A, F, G, I, L, M, P, V, W, Y) are shown in white/grey, polar residues in bright/dark green (H, N, Q, S, T), acidic residues in orange/red (D, E) and positively charged amino acids (K, R) in cyan/blue. Darker colors mark the contact interface. The surface of ActR-IIB_ECD _(right) is color coded identically. **(c) **Contact scheme of the wildtype BMP-2:ActR-IIB_ECD _interaction. Intermolecular van der Waals contacts (cutoff 4.5 Å) are marked by lines, H-bonds are shown in red. Contacts involving hydrophobic residues of BMP-2 are shown in the left panel, interactions involving polar residues of BMP-2 are on the right. The surface area (Å^2^) buried upon complex formation is indicated by small numbers.

The β-strands 3, 4 and 6 of ActR-IIB together with β6 and β7 of BMP-2 form the major part of the binding interface (Fig. 4a). A part of the loop of finger 2 of BMP-2 also contacts ActR-IIB. In total, 19 residues of BMP-2 and 17 residues of ActR-IIB (Fig. 4b,c) contribute significantly to the interface. Remarkably, in comparison with the ternary complex of BMP-2 bound to BMPR-IA and ActR-II the backbone conformation of the bound ActR-IIB and ActR-II is highly similar, exhibiting an r.m.s. deviation for the Cα-atoms of 0.6 Å (a global fit on all 558 Cα-atoms yields an r.m.s. deviation of 1.13 Å). The backbone as well as the sidechain conformation of residues in the β2β3 loop (also referred as M-loop) of ActR-IIB and ActR-II, which is possibly generating ligand specificity, are almost identical in all BMP-2 ligand-receptor ternary complexes.

The type II receptor binding site of BMP-2 exhibits a convex shape with no deep pockets and is predominantly hydrophobic (Fig. 4b). The hydrophobic amino acids are in the center and surrounded by a ring of polar and charged residues. The core interface residue of ActR-IIB, i.e. Trp60, Tyr42 Leu61, Lys56, V55, and Val81, exhibit large accessible surface areas in the unbound state, which become buried by 60 to 100% upon complex formation (Fig. 4b,c). The knuckle epitope of BMP-2 has a complementary hydrophobic surface with a horseshoe-like form (Fig. 4b). The shape results from the central Ser88, which is embedded, in the hydrophobic patch together with the peripheral polar side chain of Asn102.

In the complex Trp60 of ActR-IIB extends into a shallow pocket formed by Ala34, Pro35, Ser88, Leu90 and Leu100 of BMP-2 (Fig. 4b,c), all of which were identified as important determinants for type II receptor binding before [28]. The hydrophobic area around Trp60 measures 12 by 12 Å and is devoid of any water molecules. To test the hypothesis that the majority of the binding free energy originates from hydrophobic interactions, we have used isothermal titration calorimetry. The enthalpy ΔH, which is a measure for polar interactions, is rather small for the BMP-2:ActR-IIB_ECD _interaction (14 kcal mol^-1 ^at 25°C), suggesting that polar interactions play a minor role for the binding affinity of BMP-2 to type II receptors. In comparison, the enthalpy for the interaction of BMP-2 and BMPR-IA_ECD _is more than twice as large (-36 kcal mol^-1^). Another indication is in the temperature dependency of the enthalpy, the heat capacity change ΔC_p _which should have a negative value for interactions driven by the burial of hydrophobic interfaces [49]. As a matter of fact a rather large negative value of -496 cal mol^-1 ^K^-1 ^was determined for the ΔC_p _(see Additional file 5) of the BMP-2:ActR-IIB_ECD _interaction being consistent with the hypothesis that hydrophobic forces drive the binding of the type II receptors to BMP-2.

**Figure 5:**
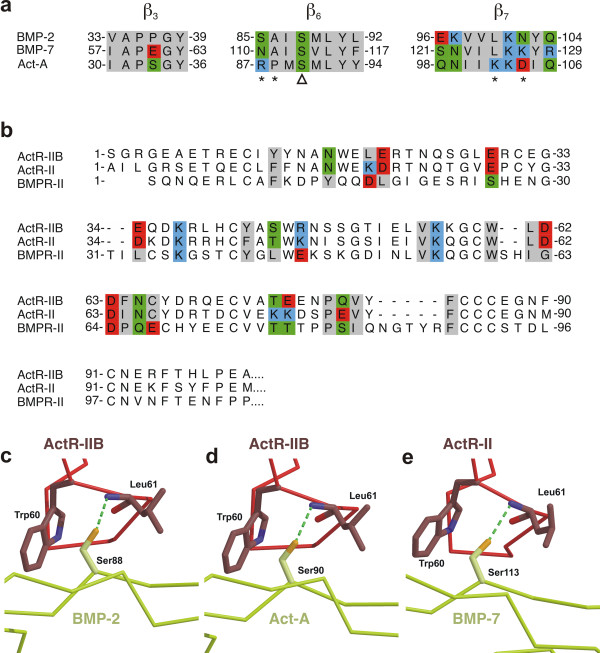
**Binding epitopes of BMPs and activin for interaction with activin receptors are very similar**. **(a) **Structure based sequence alignment for the regions of BMP-2, BMP-7 and Act-A building the knuckle epitope. The putative contact residues based on the BMP-2:ActR-IIB interaction are color coded according to Fig. 4b. Asterisks mark the amino acid positions chosen for „domain swapping" between BMP-2 and Act-A, the conserved Ser is indicated by a triangle. **(b) **Sequence alignment of the extracellular domain of ActR-IIB, ActR-II and BMPR-II, the residues contributing to the binding epitope (based on the BMP-2:ActR-IIB interface of this study) are color coded on amino acid properties as in Fig. 4b. **(c-e) **Comparison of the structural environment around the central H-bond in the complexes of **(c) **wildtype BMP-2:BMPR-IA:ActR-IIB, **(d) **Act-A:ActR-IIB (PDB entry 1S4Y, [42]) and (e) BMP-7:ActR-II (PDB entry 1LX5, [40]).

Analysis of the contact between ActR-IIB_ECD _and wildtype BMP-2 indeed yields only five intermolecular polar bonds, one ion pair and four H-bonds (see Additional file 4, Fig. 4c). It is interesting to note that in the ternary complex of BMP-2 bound to BMPR-IA and ActR-II just two H-bonds, between BMP-2 Ser88 Oγ and ActR-II Leu61 NH and between BMP-2 Glu109 Oε2 and ActR-II Lys37 Nζ, are observed [43]. This observation suggests that for low-affinity binding polar bonds contribute only marginal to the overall binding energy. The H-bond between BMP-2 Ser88 Oγ and ActR-IIB Leu61 NH is in the center of the otherwise hydrophobic epitope, whereas the other polar bonds reside at the periphery. As noted before [43], the contact residues involved in the type II receptor interactions are very similar among BMPs and activins. The horseshoe-like hydrophobic core of the knuckle epitope of BMP-2, BMP-7 and Act-A contains identical or isofunctional residues at equivalent positions (Fig. 5a). Remarkably, the polar Ser88 at the center of the epitope is highly conserved. Pronounced differences exist at the periphery of the epitope (e.g. residues at positions corresponding to residues 36, 85, 96, 97, 100, 102, 104, and 109 in BMP-2). In particular, the residues at the polar opening of the hydrophobic core and juxtaposed to conserved Ser88 vary among the BMPs and Act-A (Fig. 5a).

A comparison of the receptors ActR-IIB and ActR-II shows that the same amino acid types form the hydrophobic core around Trp60 (Fig. 5b) with 56% of the hydrophobic interface residues being identical. Most significantly, the central H-bond between receptor Leu61 (amide) and ligand Ser88 (hydroxyl group) seems to be formed in all BMP/Activin receptor complexes analyzed so far [40-43], irrespective whether ActR-IIB or ActR-II is present (Fig. 5c–e). The structural data indicate that activin type II receptor binding at the knuckle epitope is astonishingly similar in the BMPs and activins. Thus, the few side chains that differ in the ligand proteins must generate the differences observed in the relative binding affinities of the various receptor-ligand pairs.

### A hydrophobic hot spot dominates the promiscuous ligand-type II receptor interaction

In order to define the binding determinants of ActR-IIB for the BMPs and Act-A an alanine scanning analysis was performed. Interaction analysis by plasmon resonance spectroscopy employing immobilized ligands confirmed that ActR-IIB_ECD _binds Act-A with high affinity, whereas binding to BMP-2 and BMP-7 had a 50 to 100-fold lower affinity (Table 1). In the setup used in this study the apparent *K*_D _values were higher by at least two orders of magnitude than those previously determined for ActR-II/IIB using the same technique but with immobilized receptors [28,40,42] or radioligand binding to receptors in whole cells [50]. A possible explanation is due to the fact that two immobilized receptors may bind together to a single dimeric ligand thereby increasing the apparent affinity by avidity effects. This avidity effect has been quantified in the BMP system in part previously and seems to be highly dependent on the setup conditions [40]. An immobilized ligand, however, binds two single receptor ectodomains independently thereby resulting in the lower affinity of a 1:1 interaction [51], but due to the absence of any avidity effects yields more comparable data. Nevertheless the term apparent *K*_D _was used throughout since the absolute values of the *K*_D _still depend on the evaluation routine, immobilization level, and also on the method used for immobilization. This might explain the higher values reported for the affinities of the Act-A:ActR-II [40] and Act-A:ActR-IIB [42] interaction reported previously. When the low-affinity binding to BMP-2 is analyzed, the by far most disruptive mutation exists in the ActR-IIB variant W60A (Table 1). A considerable loss of affinity is also observed for the variant Y42A. Substitution of other hydrophobic residues by alanine yielded variants with a less than 10-fold loss in binding affinity for BMP-2. Substitutions at the polar residues all have no (Glu34, Gln80) or only a minor impact (Lys37, Arg46, Lys56, Asp63) on binding affinity for BMP-2. Therefore the functional binding epitope of ActR-IIB for BMP-2 seems to be dominated by Trp60 and to a lesser extent by Tyr42, which together form the hydrophobic core of the epitope. The surrounding hydrophobic side chains seem to be binding determinants of secondary importance. Four of the polar side chains (Glu34, Lys37, Arg46, and Gln80) forming ion pairs or H-bonds with BMP-2 (Fig. 4c) could be removed without major effect on affinity. Remarkably, a L61P variant has the same affinity for BMP-2 as L61A, even though the central hydrogen bond involving the amide of L61 is blocked by the proline substitution (Table 1). Therefore, the conserved central H-bond is also non-functional (silent) in the low-affinity BMP-2:ActR-IIB interaction. Together these data indicate that binding free energy of low-affinity BMP-2:ActR-IIB interaction is derived almost exclusively from hydrophobic interactions.

**Table 1:** Mutational analysis of the ActR-IIB interface (Biosensor analysis)

	Ligand proteins (immobilized)		
ActR-IIB_ECD_	BMP-2^a^	BMP-7	Act-A

	rel. *K*_D _(app. *K*_D _in nM)		

wildtype	1 (7700)	1 (3600)	1 (65)
E34A	1	1.8	1
K37A	6.1	0.3	0.7
Y42A	61	49	290
R46A	8.4	3.3	2.9
K56A	11	14	2.7
W60A	n.b^b^	n.b.	n.b.
L61A	6.4	6.4	24
L61P	7.9	19	2510
D63A	9.6	6.7	8.6
Q80A	1.3	1	1.1
F83A^c^	n.b.	n.b.	n.b.

a) For these measurements glycosylated BMP-2 expressed in CHO cells was used.

b) Binding is below detection limit (*K*_D _> 1 mM.)

c) This variant might be not native and exhibits an altered electrophoretical mobility in SDS-PAGE under non-reducing conditions.

From the functional data the binding mechanism of ActR-IIB to BMP-2 or BMP-7 seems comparable (Table 1). The main binding determinant for BMP-7 is also Trp60 followed by Tyr42. Several variants show a similar loss in affinity for BMP-2 and BMP-7. Small differences exist for the variants R46A and L61P. A significant difference is in the interaction of both BMPs with Lys37 of ActR-IIB. The ActR-IIB variant K37A binds with increased affinity to BMP-7, whereas the affinity of K37A for BMP-2 drops. Modeling studies suggested that Lys37 of ActR-IIB might be one of the key residues for generating ligand specificity among different BMP members [43]. These results obtained for BMP-7 support the hypothesis that the low-affinity binding to BMPs is determined by a hydrophobic ActR-IIB binding epitope employing virtually the same hydrophobic binding determinants.

The functional epitope of ActR-IIB for high-affinity interaction with Act-A uses the same hydrophobic main binding determinant as for binding of the BMPs. As compiled in table 1, Trp60 and Tyr42 are also crucial for binding of Act-A. The minor hydrophobic and polar determinants are of comparable importance for binding to BMPs and Act-A. Tyr42 and Leu61 seem to contribute somewhat more to Act-A than to BMP binding, whereas Lys37 and Arg46 play a likewise lesser role in Act-A binding. These small differences might contribute to ligand discrimination to some extent. However, it is probably safe to conclude that an increment in binding free energy corresponding to a low-affinity interaction is the same in binding of BMPs and Act-A, and likely the determinants for this low-affinity interaction are the same for these ligands. But in addition a further hot spot of binding is present.

A clear and probably crucial distinction of the functional Act-A binding epitope is the importance of the Leu61 amide group as revealed by comparison of the L61A and L61P variants (Table 1). The removal of the amide proton by the proline substitution leads to a 100-fold higher decrease in binding affinity (2500-fold) than the loss of the Leu61 side chain in the L61A variant (24-fold). As mentioned above BMP-2 and BMP-7 binding affinity is similar for both variants. This finding strongly suggests that the central conserved hydrogen bond, which is inactive/silent in BMP-2 and BMP-7 interaction, is active in the interaction with Act-A (Fig. 5c–e). This "switchable" H-bond seems to be responsible for the high-affinity binding of Act-A.

In conclusion, the determinant for the high-affinity binding of Act-A seems to reside in the H-bond provided by the Leu61 amide proton (which is removed in the variant L61P), whereas this central H-bond interaction does not play an important role for low-affinity interactions with the ligands BMP-2 and BMP-7.

### A switch in BMP-2 for low- and high-affinity binding of ActR-IIB

To investigate the mechanism of the ligand-specific properties of this central H-bond in more detail, residues in the BMP-2 knuckle epitope were subjected to mutational analysis (Table 2). The conserved Ser88 (Oγ), the H-bond acceptor, is surrounded by residues differing between BMP-2, BMP-7 and Act-A (Fig. 5a). We therefore performed a partial "domain swapping" by exchanging residues of BMP-2 at the positions 85, 86, 100, and 102 for those of Act-A (Table 2). BMP-2 Ser85 and Ala86 are not critical for ActR-IIB binding as seen by the *K*_D _values of the respective single and double mutants. The effect of the BMP-2 mutation S85R on the binding to ActR-II has been analyzed previously [43]. The authors report an increase in binding affinity, i.e. 2-fold. The differences in the binding affinities might be due to the different experimental setup.

**Table 2:** Type II receptor specificity of wildtype BMP-2 and variants (Biosensor analysis)

	Receptor ectodomain proteins		
BMP-2 (immobilized)	ActR-IIB	ActR-II	BMPR-II

	rel. *K*_D _(app. *K*_D _in nM)		

wildtype^a^	1 (2700)	1 (5500)	1 (24000)
S85R	1.3	0.85	0.12
A86P	2.6	1.5	0.50
S85R/A86P	1.4	1.3	0.19
L100K	0.15	0.84	1.3
N102D	7.8	3.6	0.96
L100K/N102D	0.05	0.29	0.92
Act-A^b^	0.02 (60)	0.04 (230)	0.36 (8700)
BMP-7^b^	1 (2800)	0.09 (430)	1.3 (32000)

a) BMP-2 variants were expressed in *E. coli*. Note, that the wildtype BMP-2 expressed in *E. coli *has a higher affinity to ActR-IIB than the glycosylated BMP-2 produced in CHO cells (see Table 1)

b) the relative binding constant (normalized to *E. coli *BMP-2 as a reference) of BMP-7 and Act-A are given for comparison.

The side chains at positions 100 and 102 exert a decisive role in affinity determination. BMP-2 variants containing the substitution L100K show a six to 20-fold increase in binding affinity for ActR-IIB. The BMP-2 double variant L100K/N102D binds to ActR-IIB with an affinity (*K*_D _~ 140 nM) almost as high as for the interaction of Act-A with ActR-IIB (*K*_D _~ 60 nM) (Table 2). The single mutation N102D in BMP-2 is deleterious but in combination with the exchange L100 K the affinity for ActR-IIB is further increased. The exchange of these two residues of BMP-2 therefore converts the low- into a high-affinity binding epitope and the lysine side chain seems to be the key determinant for this transformation.

**Figure 6:**
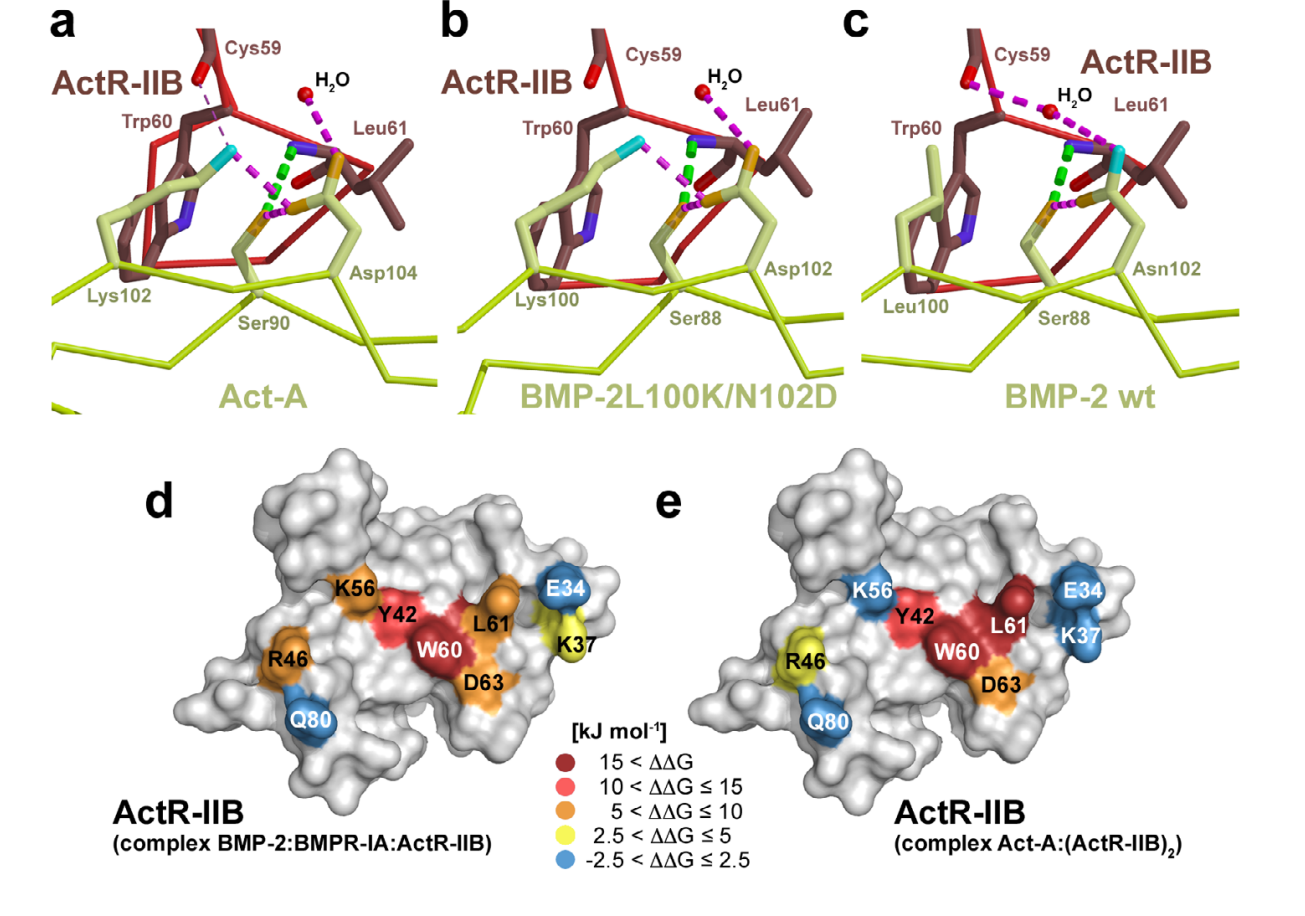
**Mechanism for affinity switch in BMP-2 L100K/N102D**. H-bond network around the conserved serine residue in Act-A (**a**), the BMP-2 variant L100K/N102D with increased ActR-IIB affinity (b) and wildtype BMP-2 (**c**). The conserved central H-bond between Ser88 Oγ (Ser90 in Act-A) and Leu61 amide of ActR-IIB is shown as green thick stippled line. The intramolecular H-bond network comprising Lys100, Asp102, Ser88 (Lys102, Asp104 and Ser90 in Act-A) and a nearby structurally conserved water molecule is indicated by stippled lines in magenta. The putative intermolecular H-bond between Lys102 of Act-A and Cys59 backbone carbonyl of ActR-IIB in the structure of the complex Act-A:ActR-IIB_ECD _(PDB entry 1S4Y, [42]) is indicated by a thin line (**a**), as this H-bond is only present on one half of the dimeric complex and its geometrical parameters are close to exclusion cutoff criteria. A comparison of the position of the structurally conserved water molecule in the three structures shows that in wildtype BMP-2 (**c**) this solvent molecule is located directly above the central H-bond indicating direct accessibility of the H-bond by solvent. (**d, e**) Surface representation of ActR-IIB color coded by the contribution (ΔΔG in kJ mol^-1^) of each residue side chain to the binding free energy for (**d**) wildtype BMP-2 and (**e**) Act-A as measured by alanine scanning mutational analysis (see Table 1). For residue L61 the exchange to proline was used to point out the influence of the central conserved H-bond. The ΔΔG values are given in kJ mol^-1^. Dark red color marks hot spots of binding with an energy contribution of more than 15 kJ mol^-1^. Residues in yellow contribute only little; energy contribution of residues marked in blue is considered insignificant.

A previous mutational analysis of Act-A has shown that a positively charged residue at position 102 (equivalent to position 100 in BMP-2) is required for activin receptor binding [52]. This indicates that this lysine is also essential for high-affinity binding of Act-A. From structure-/function analyses of the Act-A:ActR-IIB interaction it has been proposed that an intermolecular H-bond between the Lys amine group and the backbone carbonyl of Cys59 of ActR-IIB contributes to high-affinity binding (*trans *effect) [41], although the geometrical parameters of this H-bond are close to the exclusion criteria (Fig. 6a). A similar mechanism has been also proposed from the structure analysis of BMP-2 bound to BMPR-IA and ActR-II [43]. A comparison of the binding affinities of ActR-IIB variants to Act-A and BMP-2 already suggested that the main difference is, however, in the strength of the central conserved H-bond between Ser88 Oγ (BMP-2) and Leu61 amide (ActR-IIB). Confirming this, our structure of the ternary complex (1:2:2) comprising BMP-2L100K/N102D shows no additional intermolecular H-bond between the lysine side chain and Cys59 of ActR-IIB (Fig. 6b).

Instead a double mutant cycle (Table 3) now reveals that the strength of the H-bond between Ser88 Oγ (BMP-2) and Leu61 amide (ActR-IIB) can be modulated through the presence of a Lys and an Asp residue at position 100 and 102. Disruption of the central conserved H-bond, i.e. by the ActR-IIB variant L61P, in the environment of Lys100 and Asp102 leads to a drastic reduction in binding affinity to BMP-2 similar to that observed for the interaction Act-A:ActR-IIBL61P. Comparing the affinities of the ActR-IIB variants L61P and L61A to wildtype BMP-2 shows no difference, indicating that, by contrast, in the wildtype environment the central H-bond does not contribute to the binding free energy (Table 3, Fig. 6d,e).

**Table 3:** Double mutant cycle of BMP-2 and ActR-IIB_ECD _(Biosensor analysis)

	Receptor ectodomain proteins		
BMP-2^a ^(immobilized)	ActR-IIB	ActR-IIBL61A	ActR-IIBL61P

	app. *K*_D _in Nm		

wildtype	2700	42000	76000
L100K/N102D	140	14000	160000
Act-A^b^	60	2000	180000

a) BMP-2 variants were expressed in *E. coli*.

b) the apparent binding constant of Act-A is shown for comparison.

The residues Lys100 and Asp102 are located directly above the intermolecular H-bond between BMP-2 Ser88 Oγ and ActR-IIB Leu61 amide at the opening of the horseshoe-like hydrophobic core of the epitope (Figs. 4b and 6a,b). In the structures of the Act-A:ActR-IIB_ECD _[41] and the ternary complex (1:2:2) of BMP-2L100K/N102D the Lys and Asp residues form a stable intramolecular salt bridge (Fig. 6a,b). Accordingly, Lys100 and Asp102 (in BMP-2L100K/N102D) exhibit low temperature factors indicating that both residues are quite immobile. In the structure of the ternary complex (1:1:1) comprising wildtype BMP-2 the temperature factors of the equivalent residues Leu100 and Asn102 are higher than for nearby residues indicating increased flexibility. The "rigid lid" centered above the conserved H-bond between BMP-2 Ser88 Oγ and ActR-IIB Leu61 amide might provide an effective shielding from solvent access thereby enhancing the strength of this particular H-bond (Fig. 6a–c). By thus modulating the energy contribution of an existing interaction (*cis *effect) rather than adding a new (*trans *effect) one achieves the switch from low- to high-affinity.

### Specificity in the BMP-2 type II receptor epitope

Individual and combined mutations at BMP-2 position 85, 86, 100 and 102 influence binding of unalike type II receptors (Table 2) suggesting that these residues determine binding specificity of BMP-2 for ActR-II, ActR-IIB and BMPR-II. The exchange of Leu100 of BMP-2 to lysine specifically increases the binding affinity for ActR-IIB, whereas binding to ActR-II and BMPR-II is basically unaffected. The double variant L100K/N102D shows a large increase in affinity for ActR-IIB, in addition the affinity for ActR-II is moderately increased (threefold), but binding to BMPR-II remains unaltered. Mutating residues Ala86 and in particular Ser85 in BMP-2 results in variants that exhibit an increased binding affinity exclusively for BMPR-II whereas binding to the activin type II receptors is either unaffected or even decreased. The specific effects of these mutations indicate that the BMP-2 knuckle epitope comprises single residues specifying relative affinities and therefore specificity for the interaction with type II receptors ActR-IIB, ActR-II and BMPR-II (Fig. 5a,b).

**Figure 7:**
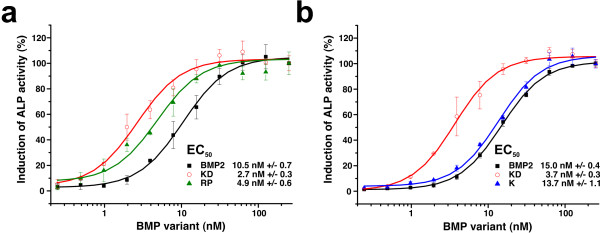
**Biological activities of specificity-altered BMP-2 variants**. (**a**) The BMP variants L100K/N102D (red) and S85R/A86P (green) exhibit a two- to three-fold lower EC_50 _value for ALP induction as wildtype BMP-2. The increased biological activity correlates with the increased affinity for ActR-IIB/ActR-II (L100K/N102D) or BMPR-II (S85R/A86P). (**b**) The single variant BMP-2 L100K, which has a six-fold higher affinity for ActR-IIB, shows no increased biological activity in C2C12 cells.

In order to understand if the change in type II receptor specificity of these BMP-2 variants is reflected in their biological activity, the variants were tested for their capabilities to induce alkaline phosphatase (ALP) expression in the myoblast cell line C2C12 (Fig. 7). The C2C12 cells express the type II receptors ActR-II and BMPR-II but not ActR-IIB [53]. The two BMP-2 variants S85R/A86P and L100K/N102D have a three to four-fold lower EC_50 _value than wildtype BMP-2 corresponding to a higher biological activity (Fig. 7a). These results seem to reflect the three to five-fold higher affinity of the double mutants for the ActR-II, respectively the BMPR-II receptor. In contrast, when the single mutant L100K is analyzed in parallel with wildtype BMP-2 the EC_50 _is the same (Fig. 7b). This might be expected since the BMP-2 variant L100K has an unaltered affinity for ActR-II and BMPR-II and the specifically increased affinity of L100K for ActR-IIB is biologically not relevant in this specific cell type. In summary, these data strongly suggest that the specific alterations in the mutant BMP-2 can be biologically relevant.

## Discussion

ActR-IIB is one of three type II receptors known to interact with BMP-2 [15]. The ectodomains of all three type II receptors bind with low affinity to this ligand (Table 2). BMPR-II has the lowest affinity although in many cells it is the prototypic type II receptor for BMP-2. The affinity of ActR-IIB, the strongest BMP-2 binder, is about 10-fold higher. In how far these differences in low-affinity type II binding are important for BMP-2 signaling is unknown. The increase in the biological activities observed for BMP-2 variants in our study suggests that such small differences might be of functional relevance.

As shown by cross-linking experiments in whole cells, low-affinity type II receptors alone do not bind or bind only weakly to solute BMP-2 [36,54,55]. On the other hand BMPR-IA, the high-affinity receptor of BMP-2, is efficiently cross-linked when present alone, and in its presence cross-linking of the type II receptor proceeds efficiently [54,55]. The BMPR-IA ectodomain binds BMP-2 in our experimental setup with a dissociation constant of 10 to 20 nM. This affinity is 100 to 200-fold higher than that of the type II chains. On the basis of this large difference in the relative affinities of the type I and type II receptors a 2-step assembly mode of the ternary complex can be postulated, where solute BMP-2 binds first to the high-affinity BMPR-IA receptor and subsequently in the membrane the binary BMP-2/type I receptor complex can associate with the type II receptor [48].

The affinity profile of Act-A for the type II ectodomains is completely different from BMP-2. In the present experiments the apparent *K*_D _of Act-A for ActR-IIB is about 60 nM (Act-A to ActR-II *K*_D _~ 250 nM), typical for a high-affinity interaction. In contrast BMPR-II_ECD _is bound by Act-A with low affinity (*K*_D _~ 10 μM) resembling the interaction with BMP-2. The type I activin receptor ActR-IB by itself has an exceedingly low affinity for Act-A [14]. Chemical cross-linking in the membrane is therefore only possible in the presence of the high-affinity ActR-II/B receptors [56]. Consequently, the assembly of the ternary activin receptor complex differs in crucial aspects from that of the BMP-2 receptor. Solute Act-A binds first to its high-affinity ActR-II/B receptors before the low-affinity ActR-IB receptor is recruited into the complex in the membrane [56]. As a consequence the type II activin receptors play different roles and are involved in different assembly modes during BMP-2 versus activin receptor activation. In this context BMPR-II is a special case. It likely cannot signal with Act-A, since it has only a low affinity for this ligand and a high-affinity type I chain is unknown. This generally low affinity of BMPR-II for BMPs and activin possibly explains why cytoplasmatically truncated BMPR-II exerts a dominant negative effect on BMP but not on activin signaling [57]. The truncated BMPR-II can compete with the low-affinity interaction of type II receptors and BMP-2 but not with the high-affinity interaction of ActR-II/B and activin.

The ectodomains of the ActR-II and ActR-IIB share 44% sequence identity (Fig. 7b). The affinities of BMP-2 for the two isoforms are similar (see Table 2, [40]). It is therefore interesting to compare the interface of BMP-2 for ActR-IIB (this study) and ActR-II [43]. The hydrophobic core around Trp60 (ActR-IIB numbering) is remarkably conserved. Only residues Tyr42 and Tyr13 are replaced by phenylalanine residues in ActR-II, however since both tyrosine residues are no involved in H-bonds the replacement should be without affect for the binding affinity. Polar bonds at the periphery of the epitopes are different supporting the conclusion already drawn from the mutational analysis of ActR-IIB that they are not important for binding affinity. Only the central H-bond connecting BMP-2 Ser88 Oγ and Leu61 amide also exists in the complex with ActR-II. Since a BMP-2 S88A variant exhibits only a small decrease in binding affinity for ActR-II [28] it can be assumed that this H-bond is silent or very weak as in the complex of BMP-2 with ActR-IIB. In this context it is intriguing that the BMP-2 variants L100K and L100K/N102D have in comparison to ActR-IIB only a small effect on binding affinity for ActR-II. In particular the double variant L100K/N102D conferring high-affinity binding to ActR-IIB has a six-fold lower effect on ActR-II binding, while the single variant L100K shows no increase in affinity for ActR-II at all (Table 2). The *K*_D _value for the interaction between ActR-II and BMP-2 L100K has been reported before [43] to be five to eight-fold lower than for wildtype BMP-2. The reason for the discrepancy to the present results is as yet unclear. BMP-2 L100K/N102D has not been structurally analyzed in complex with ActR-II so far. Possibly, the intramolecular hydrophobic lid formed by Lys-Asp pair is less efficient in complex with ActR-II. Considering the identical side chain composition at the core of the receptor epitopes it would be surprising, however, if here an intermolecular H-bond between Lys102 of the BMP-2 variant and the backbone carbonyl of ActR-II Cys59, which is not observed in the complex with ActR-IIB, would contribute to binding affinity [43].

Previously, BMP-2 mutants have been generated, which function as BMP antagonists either due to disruption of the knuckle epitope [28] or as Noggin-blocker due to inactivation of the wrist epitope [48]. Now new BMP-2 variants could be obtained with increased biological activity resulting from improved binding to ActR-II (L100K/N102D) or BMPR-II (S85R/A86P) (Table 2). It will be interesting to study if the "superagonist" activity of these mutant proteins will be retained *in vivo*, e.g. in an ectopic bone formation assay or during healing of a critical size bone defect. BMP-2 L100K/N102D may also function as an ActR-IIB blocker, even though its affinity for this receptor is still not as high as that of Act-A (Table 2) or of GDF-8/-11 (W. Sebald, unpublished). However, it is unclear whether BMP-2 variants can interact with the type I receptor ActR-IB to some extent when strongly bound to ActR-IIB. The results of the present structure/function analysis of ActR-IIB might give some clues how relative affinity/specificity to certain ligands can be mutationally altered and manipulated. This might become useful for the design of receptor ectodomain constructs, which can specifically inhibit certain BMPs, GDFs, activins, or other ligands of the TGF-β superfamily.

## Conclusion

In this study we present a detailed structure-function analysis of the interaction of BMP-2 – a prototypic ligand of the TGF-β superfamily – with its type II receptor ActR-IIB. In previous work the determinants for specificity and affinity in type I BMPR-IA and BMPR-IB receptor interaction have been analyzed [32,33]. Now new crystal structures of ternary complexes comprising BMPR-IA and ActR-IIB ectodomains and either wildtype BMP-2 or a BMP-2 variant with enhanced affinity for ActR-IIB have been elucidated at high resolution. On the basis of the structural information a mutational analysis of ActR-IIB and the interacting BMP-2 knuckle epitope has been performed in order to investigate possible interactions between epitopes as well as determinants for specificity and affinity in type II receptor interaction, in particular with the dual-specificity receptor ActR-IIB. The results reveal a molecular basis for understanding differences in BMP-2, BMP-7 and Act-A signaling. In addition the molecular mechanisms for high- and low-affinity binding to the ActR-IIB receptor have been clarified. The present results will possibly help to design ligand and receptor mutant proteins that can be used to target diseases caused by dysregulation of BMP or activin signaling.

## Methods

### Expression and purification of recombinant proteins

*E. coli *derived BMP-2 proteins and the receptor ectodomain proteins of BMPR-IA, ActR-II and BMPR-II were expressed and purified as described earlier [28]. The ActR-IIB_ECD _was expressed as thioredoxin-fusion similar to BMPR-IA_ECD _in OrigamiB (DE3) (Novagen) cells. Cells were harvested and lysed by sonication. The fusion protein was isolated from the supernatant by Ni^2+^-IMAC chromatography, cleaved with 0.5 U thrombin per mg fusion protein (Sigma) and the products subsequently purified by anion exchange chromatography (TMAE, Merck). ActR-IIB_ECD _was finally purified by reversed phase HPLC using a C4 column (Vydac). BMP-2 or ActR-IIB_ECD _variants were constructed by site directed mutagenesis using the QuikChange methodology (Stratagene).

For acquisition of a MAD dataset using Se-Met derivatives, a triple mutant variant of BMPR-IA_ECD _(F35M, L73M, L95M) was generated. Binding properties to BMP-2 were unaltered as tested by interaction analysis. The variant and wildtype BMP-2 were expressed in M9 minimal medium supplemented with 50 mg L^-1 ^DL-Se-Met (Sigma) using the Met-auxotroph *E. coli *strain B834(DE3) (Novagen).

### Crystallization of the ternary ligand-receptor complex

The ternary complexes consisting of BMP-2 or BMP-2 L100K/N102D bound to BMPR-IA_ECD _and ActR-IIB_ECD _were prepared by a stepwise procedure. First the binary BMP-2:BMPR-IA_ECD _complexes were formed and purified as described [58]. The purified binary complexes were then mixed with a 2.2-fold molar excess of ActR-IIB_ECD _in HBS_700 _buffer (10 mM HEPES, 700 mM NaCl pH7.4) and purified by gel filtration. Stoichiometry and homogeneity of both complexes were analyzed by comparison to mixtures of the components with defined molar ratios using SDS-PAGE and RP-HPLC. For crystallization the protein complexes were concentrated to 15 mg ml^-1^. Crystals of wildtype BMP-2: BMPR-IA_ECD_:ActR-IIB_ECD _were obtained by hanging-drop vapor diffusion from 50% (v/v) PPG400, 0.1 M Bis-Tris, pH5.8 at 21°C; for the complex comprising the BMP-2 double variant crystallization was achieved from 30% (w/v) PEG3350, 0.1 M Tris-HCl pH8.8, 0.2 M ammonium acetate at 21°C. Crystals of a final size of approximately 250 × 100 × 100 μm grew within 8 days for both complexes.

### Data acquisition and structure analysis

A MAD dataset of the ternary complex (1:1:1) was acquired at three wavelengths (inflection, peak, remote) with 360° rotation of the crystal (1° per frame) were measured; a maximum resolution of 2.7 Å was obtained by recording with 30 s exposure time per degree (beamline BL14.1 at BESSY, Protein Structure Factory, Germany). The data were processed and integrated using MOSFLM version 6.2.1, scaling was performed using SCALA CCP4 version 4.2.1 (see Additional file 6). The positions of the Selen-sites were determined using SHELX, and refined using SOLVE version 2.06. RESOLVE and ARP/WARP were used to automatically trace the electron density and to yield an initial model. The structures of BMP-2 monomer, BMPR-IA (both PDB 1REW, [48]) and ActR-IIB (PDB 1NYS, [41]) were superimposed onto tracing models to facilitate further model building. High-resolution native data for both ternary complexes were acquired at 100 K on a home-source consisting of a Rigaku MicroMax007, Osmic mirror optics and a Rigaku R-AXIS IV++ detector. The exposure time for crystals of the ternary complex (1:1:1) was 750 s per 0.5° rotation, crystals diffracted up to 1.75 Å. A complete dataset was obtained from an 85° sweep, processing was performed using the software CrystalClear (Rigaku Inc.) (Table 4). The initial model of the MAD dataset was used to interpret and refine the high-resolution data acquired for the ternary complex (1:1:1). Data for the ternary complex (1:2:2) were also acquired on the above described home source, the exposure time was 300 s per 0.5°, crystals diffracted up to 1.8 Å. A complete dataset was acquired from a sweep of 129°. The data were interpreted by molecular replacement using the software Phaser employing the components of the ternary complex (1:1:1) structure as search models.

**Table 4:** Processing and refinement statistics

Data processing	ternary complex 1:1:1	ternary complex 1:2:2
Space group	P2_1_2_1_2_1_	P3_2_21
Unit cell	a = 64.1 Å, b = 65.4 Å, c = 114.1 Å α = β = γ = 90°	a = b = 82.8 Å, c = 111.1 Å α = β = 90°, γ = 120°
Resolution (Å)	20.0–1.81 Å (1.89–1.81 Å)	41.4–1.78 Å (1.84–1.78 Å)
Wavelength	1.5418 Å	1.5418 Å
Number of measured reflections^c^	134179 (11577)	282925 (18238)
Number of unique reflections^c^	43615 (4532)	42606 (4140)
Completeness	98.6 (93.7)%	99.6 (98.7)%
Multiplicity	2.9 (2.6)	6.6 (4.4)
R_sym _for all reflections	5.5 (38.9)%	6.9 (51.4)%
<Intensity/σ>	9.6 (2.5)	12.6 (2.6)

Refinement statistics		

Resolution	20–1.85 Å (1.92–1.85 Å)	40–1.92 Å (1.99–1.92 Å)
R_cryst_	21.6 (36.7)%	22.8 (42.5)%
R_free _(Test set 5%)	22.5 (41.3)%	26.4 (42.5)%
r.m.s. deviation		
Bonds	0.013 Å	0.006 Å
Angles	1.572°	1.249°
Torsion angles	1.084°	0.794°
Average B-Factor	38.7 Å^2^	52.4 Å^2^
Cross-validated sigma coordinate error	0.37 Å	0.45 Å
Solvent content	52.8%	60.9%

Procheck analysis^d^		

Residues in most favored region	86.4% (291)	86.2% (219)
Residues in additional allowed region	11.9% (40)	13.0% (33)
Residues in generously allowed region	1.8% (6)	0.8% (2)
Residues in disallowed region	0% (0)	0% (0)

Statistical analysis for the highest resolution shell is shown in parentheses.

a) BMP-2:BMPR-IA_ECD_:ActR-IIB_ECD _complex

b) BMP-2L100K/N102D:(BMPR-IA_ECD_)_2_:(ActR-IIB_ECD_)_2 _complex

c) cut-off for reflections F > 0σ

d) number of residues is shown in parentheses

### Interaction analysis by surface plasmon resonance

The BIACORE^2000 ^system was used for all biosensor experiments. For measurement of ligand-specific binding capabilities to different type II receptors a streptavidin-modified biosensor CM5 was coated with biotinylated BMP-2 (*E. coli*), BMP-2 (CHO cells, R&D Systems), BMP-7 (CHO cells, R&D Systems), or Act-A (Sf9 cells) [59] to a level of about 200 resonance units (1 RU = 1 pg mm^-2^). Interaction with the type II receptors ActR-IIB, ActR-II [28] and BMPR-II [28] was analyzed by recording sensorgrams of the ligand-receptor interactions in HBS500 buffer (10 mM HEPES, pH7.4, 500 mM NaCl, 3.4 mM EDTA, 0.005% surfactant P20) using the receptor ectodomain proteins as analyte. Surfaces were regenerated by perfusion for 2 min with 4 M MgCl_2_. All measurements were corrected for non-specific interactions by subtracting a control sensorgram recorded for flow cell 1. Apparent binding constants (*K*_D_) were obtained from the dose dependence of equilibrium binding using 1, 2, 3, 5, 10, 20, and 50 μM concentration of the receptor ectodomain proteins. The mean standard deviations for all *K*_D _values were < 20%.

### Induction of alkaline phosphatase (ALP) expression

The mouse myoblast cell line C2C12 (ATCC, No. CRL-1772) was cultured in DMEM:HamsF12 (1:1) medium containing 5% fetal calf serum (FCS), and antibiotics (100 U ml^-1 ^penicillin G and 100 μg ml^-1 ^streptomycin). For alkaline phosphatase induction (ALP) assays the cells were serum starved (2% FCS) and exposed to ligands for 72 h in 96-well microplates [60]. After cell lysis ALP activity was measured by p-nitrophenylphosphate conversion using an ELISA reader at 405 nm.

## Authors' contributions

D.W. performed the mutagenesis, protein expression and purification and crystallization of the wildtype BMP-2 ligand and receptor proteins. A.K. and U.M. participated in the data acquisition and analysis; J.N. and W.S. performed the interaction measurements and analysis. S.H. prepared, purified and crystallized the components of the ternary complex comprising the double variant BMP-2 L100K/N102D. Measurement of the biological activities of the BMP-2 variants was carried out by A.S. T.D.M. and W.S. conceived the study, wrote the manuscript and participated in all stages of the work. All authors read and approved the final manuscript.

## Supplementary Material

Additional File 1 Stoichiometry of the ternary complex comprising wildtype BMP-2, BMPR-IA_ECD _and ActR-IIB_ECD_. Analysis of the stoichiometry of the ternary ligand-receptor complex of wildtype BMP-2 using analytical SDS-PAGE and reversed-phase HPLC to confirm that the unusual stoichiometry (1:1:1 for BMP-2:BMPR-IA:ActR-IIB) observed in the crystal is not present in solution. Click here for file

Additional File 2 Comparison of the crystal structures of the ternary complexes. Superposition of the ternary ligand-receptor complexes of wildtype BMP-2 (1:1:1) and BMP-2L100K/N102D (1:2:2). Click here for file

Additional File 3 Binding of the type II receptor ectodomain is not dependent on type I receptor interaction. Possible cooperativity in the recruitment of type II receptor ectodomain due to the presence of the type I receptor ectodomain was investigated by interaction analysis (biosensor analysis) Click here for file

Additional File 4 Hydrogen bonding network in the ternary ligand-receptor complexes of wildtype BMP-2 and BMP-2L100K/N102D. Intermolecular hydrogen bonds between the ligand (BMP-2 or BMP-2L100K/N102D) and the type I receptor BMPR-IA and the type II receptor ActR-IIB Click here for file

Additional File 5 Binding of the type II receptor is dominated by hydrophobic interactions. Thermodynamic parameters of the interaction of ActR-IIB_ECD _with the binary complex BMP-2:BMPR-IA_ECD _were determined by isothermal calorimetry (ITC) Click here for file

Additional File 6 MAD data for the ternary complex (1:1:1) BMP-2:BMPR-IA_ECD_:ActR-IIB_ECD_. Acquisition and processing statistics on a 3-wavelength MAD dataset used to solve the structure of the ternary complex (1:1:1) of wildtype BMP-2 Click here for file
