# Supplementary material for: A silent H-bond can be mutationally activated for high-affinity interaction of BMP-2 and activin type IIB receptor
Source: BMC Struct Biol. 2007 Feb 12;7:6. doi: 10.1186/1472-6807-7-6 (PMC1802081; doi:10.1186/1472-6807-7-6)
Supplement: Additional File 1 — Stoichiometry of the ternary complex comprising wildtype BMP-2, BMPR-IAECD and ActR-IIBECD. Analysis of the stoichiometry of the ternary ligand-receptor complex of wildtype BMP-2 using analytical SDS-PAGE and reversed-phase HPLC to confirm that the unusual stoichiometry (1:1:1 for BMP-2:BMPR-IA:ActR-IIB) observed in the crystal is not present in solution. [file 1472-6807-7-6-S1.pdf]

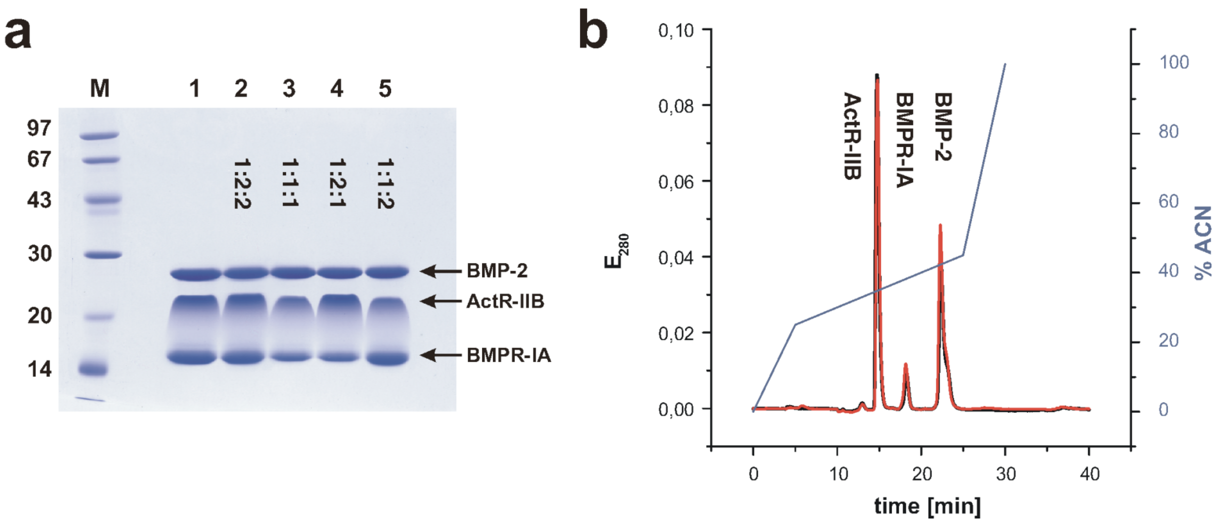

Stoichiometry of the ternary complex comprising wildtype BMP-2, BMPR-IA$_{ECD}$ and ActR-IIB$_{ECD}$

(**a**) Stoichiometry of the ternary complex comprising wildtype BMP-2, BMPR-IA$_{ECD}$ and ActR-IIB$_{ECD}$ in solution determined by analytical SDS-PAGE. Lane M denotes protein marker with the indicated molecular weights. Lane 1 is the ternary complex of wildtype BMP-2 as isolated from gelfiltration with 150pmol of complex loaded. In lanes 2 to 5 mixtures of BMP-2, BMPR-IA$_{ECD}$ and ActR-IIB$_{ECD}$ with defined molar ratios (as indicated) were loaded, e.g. 1:2:2 means 150pmol BMP-2, 300pmol BMPR-IA$_{ECD}$ and 300pmol ActR-IIB$_{ECD}$. The staining intensities of the individual protein bands were integrated using the software NIH Image J version 1.37a (http://rsb.info.nih.gov/ij/). Calculation of the molar ratios by comparing the integrated intensities of the isolated ternary complex with those of the standard mixtures yields a molar ratio of 1.0:1.9:2.1 (BMP-2 dimer:BMPR-IA$_{ECD}$:ActR-IIB$_{ECD}$). The standard deviation is 5%. (**b**) Overlay of reversed phase HPLC chromatograms of the ternary complex (comprising wildtype BMP-2) as isolated from gelfiltration (red line) and a mixture of BMP-2, BMPR-IA$_{ECD}$ and ActR-IIB$_{ECD}$ in the molar ratio 1:2:2 (black line). The gradient of acetonitrile is indicated in blue. The peaks of the chromatogram were integrated using the software Clarity Lite (Data Apex Inc.); the areas were then divided by the extinction coefficients yielding an experimental molar ratio for the isolated ternary complex of 0.98:2.13:1.97 (BMP-2 dimer:BMPR-IA$_{ECD}$:ActR-IIB$_{ECD}$). The standard deviation for the molar ratios is 10%.
