# Supplementary material for: A silent H-bond can be mutationally activated for high-affinity interaction of BMP-2 and activin type IIB receptor
Source: BMC Struct Biol. 2007 Feb 12;7:6. doi: 10.1186/1472-6807-7-6 (PMC1802081; doi:10.1186/1472-6807-7-6)
Supplement: Additional File 2 — Comparison of the crystal structures of the ternary complexes. Superposition of the ternary ligand-receptor complexes of wildtype BMP-2 (1:1:1) and BMP-2L100K/N102D (1:2:2). [file 1472-6807-7-6-S2.pdf]

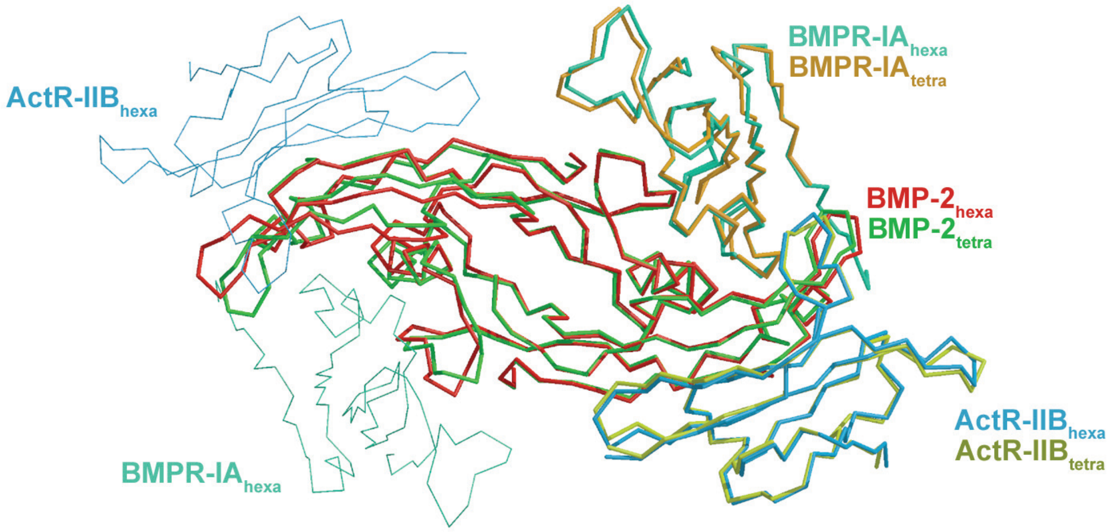

Comparison of the crystal structures of the ternary complexes

Superposition of the ternary complexes (1:1:1) (comprising wildtype BMP-2:BMPR-IA$_{ECD}$:ActR-IIB$_{ECD}$) and (1:2:2) (comprising BMP-2L100K/N102D:(BMPR-IA$_{ECD}$)$_2$:(ActR-IIB$_{ECD}$)$_2$). Fitting was performed using the positions of the Cα atoms of BMP-2 involved in ligand-receptor contacts and the receptor ectodomains. The overlay shows that the structures of both crystal forms are basically identical for the ligand-receptor interacting parts. Only the conformations of some loops in the BMP-2 monomer of the ternary complex (1:1:1) that are not involved in receptor interactions, namely the pre-helix loop in the wrist epitope, differ to the conformations of the respective regions in their bound state.
