# Supplementary material for: A silent H-bond can be mutationally activated for high-affinity interaction of BMP-2 and activin type IIB receptor
Source: BMC Struct Biol. 2007 Feb 12;7:6. doi: 10.1186/1472-6807-7-6 (PMC1802081; doi:10.1186/1472-6807-7-6)
Supplement: Additional File 3 — Binding of the type II receptor ectodomain is not dependent on type I receptor interaction. Possible cooperativity in the recruitment of type II receptor ectodomain due to the presence of the type I receptor ectodomain was investigated by interaction analysis (biosensor analysis) [file 1472-6807-7-6-S3.pdf]

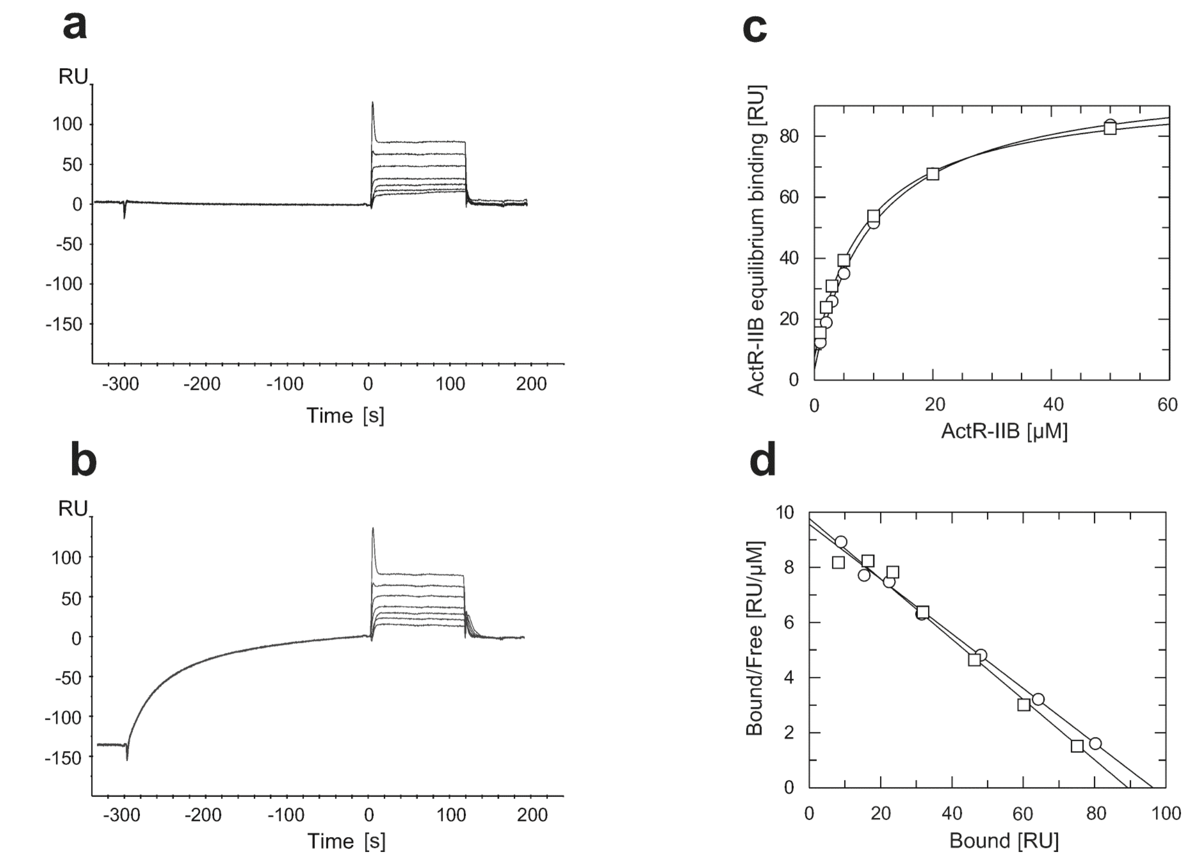

Binding of the type II receptor ectodomain is not dependent on type I receptor interaction

To measure possible cooperativity, binding of the ActR-IIB$_{ECD}$ to immobilized BMP-2 was compared to that of a binary complex consisting of BMP-2 bound to immobilized BMPR-IA$_{ECD}$. All measurements were corrected for non-specific interactions by subtracting a control sensorgram. Apparent K_D values were obtained from the dose dependence of equilibrium binding using 1, 2, 3, 5, 10, 20, and 50μM concentration of ActR-IIB ectodomain protein. Sensorgrams of the ActR-IIB$_{ECD}$ interaction with **(a)** immobilized BMP-2 and **(b)** BMP-2 in complex with immobilized BMPR-IA$_{ECD}$. At time -300s perfusion with 200nM BMP-2 was started to form the binary complex BMP-2:BMPR-IA$_{ECD}$. At time 0s ActR-IIB$_{ECD}$ was injected for 120s at above mentioned concentrations between 1 and 50μM. **(c)** The sensorgrams in (**a**) and (**b**) have been evaluated to show the dose dependency of ActR-IIB equilibrium binding to BMP-2 alone (o) and to a binary BMP-2:BMPR-IA$_{ECD}$ complex (□). **(d)** Scatchard analysis of the curves presented in c. The apparent K_D values were 10μM for BMP-2 alone (o) or 9μM for BMP-2 in complex with BMPR-IA$_{ECD}$ (□).
