# Supplementary material for: A silent H-bond can be mutationally activated for high-affinity interaction of BMP-2 and activin type IIB receptor
Source: BMC Struct Biol. 2007 Feb 12;7:6. doi: 10.1186/1472-6807-7-6 (PMC1802081; doi:10.1186/1472-6807-7-6)
Supplement: Additional File 4 — Hydrogen bonding network in the ternary ligand-receptor complexes of wildtype BMP-2 and BMP-2L100K/N102D. Intermolecular hydrogen bonds between the ligand (BMP-2 or BMP-2L100K/N102D) and the type I receptor BMPR-IA and the type II receptor ActR-IIB [file 1472-6807-7-6-S4.pdf]

**(a) Hydrogen bonding network in the type I and II ligand-receptor interface of wildtype BMP-2 bound to BMPR-IA$_{ECD}$ and ActR-IIB$_{ECD}$ (ternary complex 1:1:1)**

H-bonds between BMP-2 and BMPR-IA$_{ECD}$ in the ternary complex (1:1:1) wtBMP-2:BMPR-IA:ActR-IIB

| BMP-2 | BMPR-IA | Distance Å | Angle NOC[a] | H-bond[b] |
|---|---|---|---|---|
| Ser24$_A$ (O) | Lys92 (NZ) | 3.06 | 152 | MC-SC |
| Val26$_A$ (O) | Ser90 (OG) | 2.59 | 133 | MC-SC |
| Trp28$_A$ (NE1) | Asp89 (O) | 2.79 | 136 | SC-MC |
| Leu51$_B$ (N) | Gln86 (OE1) | 2.86 | 128 | MC-SC |
| Leu51$_B$ (O) | Gln86 (NE2) | 2.98 | 135 | MC-SC |
| Asp53$_B$ (N) | Cys77 (O) | 2.80 | 129 | MC-MC |
| Asp53$_B$ (OD1) | Lys79 (NZ) | 3.01 | 111 | SC-SC |
| Asp53$_B$ (OD2) | Thr55 (OG1) | 2.61 | 132 | SC-SC |
| Ser69$_B$ (O) | Gln94 (N) | 2.86 | 151 | MC-MC |
| Ser69$_B$ (OG) | Arg97 (NH2) | 3.07 | 92 | SC-SC |
| Ser72$_B$ (OG) | Gln94 (OE1) | 2.97 | 129 | SC-SC |
| Lys101$_A$ (NZ) | Asp84 (OD2) | 2.95 | 146 | SC-SC |
| Tyr103$_A$ (OH) | Asp84 (OD2) | 2.97 | 116 | SC-SC |

H-bonds between BMP-2 and ActR-IIB$_{ECD}$ in the ternary complex (1:1:1) wtBMP-2:BMPR-IA:ActR-IIB

| BMP-2 | ActR-IIB | Distance Å | Angle NOC[a] | H-bond[b] |
|---|---|---|---|---|
| Ala86$_A$ (N) | Glu34 (OE2) | 3.24 | 147 | MC-SC |
| Ser88$_A$ (OG) | Leu61 (N) | 2.82 | 131 | SC-MC |
| Glu96$_A$ (O) | Arg46 (NH2) | 2.96 | 171 | MC-SC |
| Glu96$_A$ (OE1) | Gln80 (NE2) | 3.05 | 147 | SC-SC |
| Glu109$_A$ (OE2) | Lys37 (NZ) | 2.62 | 140 | SC-SC |

a) N, O, C are the donor-acceptor atoms; from general statistics this angle is 149° ± 15° for MC-MC hydrogen bonds and 129° ± 18° for SC-MC and SC-SC H-bonds;

b) MC (main chain) and SC (side chain) donor/acceptor atoms;

**(b) Hydrogen bonding network in the type I and II ligand-receptor interface of the BMP-2 double variant L100K/N102D bound to BMPR-IA$_{ECD}$ and ActR-IIB$_{ECD}$ (ternary complex 1:2:2)**

H-bonds between BMP-2 and BMPR-IA$_{ECD}$ in the ternary complex (1:2:2)
BMP-2L100K/N102D:(BMPR-IA$_{ECD}$)$_2$:(ActR-IIB$_{ECD}$)$_2$

| BMP-2 | BMPR-IA | Distance Å | Angle NOC[a] | H-bond[b] |
|---|---|---|---|---|
| Val26$_A$ (O) | Ser90 (OG) | 2.88 | 128 | MC-SC |
| Trp28$_A$ (NE1) | Asp89 (O) | 2.89 | 135 | SC-MC |
| Leu51$_B$ (N) | Gln86 (OE1) | 2.83 | 123 | MC-SC |
| Leu51$_B$ (O) | Gln86 (NE2) | 3.00 | 132 | MC-SC |
| Asp53$_B$ (N) | Cys77 (O) | 2.90 | 124 | MC-MC |
| Asp53$_B$ (OD2) | Thr55 (OG1) | 2.80 | 125 | SC-SC |
| Asn59$_B$ (N) | Glu81 (OE2) | 3.00 | 127 | MC-SC |
| Asn59$_B$ (OD1) | Gly82 (N) | 3.09 | 147 | SC-MC |
| Ser69$_B$ (O) | Gln94 (N) | 2.87 | 148 | MC-MC |
| Ser69$_B$ (OG) | Arg97 (NH2) | 2.70 | 116 | SC-SC |
| Val70$_B$ (O) | Gln94 (NE2) | 2.87 | 94 | MC-SC |
| Ser72$_B$ (OG) | Gln94 (NE2) | 3.10 | 117 | SC-SC |
| Tyr103$_A$ (OH) | Asp84 (OD2) | 2.76 | 113 | SC-SC |

H-bonds between BMP-2 and ActR-IIB$_{ECD}$ in the ternary complex (1:2:2)
BMP-2L100K/N102D:(BMPR-IA$_{ECD}$)$_2$:(ActR-IIB$_{ECD}$)$_2$

| BMP-2 | ActR-IIB | Distance Å | Angle NOC[a] | H-bond[b] |
|---|---|---|---|---|
| Ser88 (OG) | Leu61 (N) | 2.90 | 128 | SC-MC |
| Glu96 (O) | Arg46 (NH2) | 3.24 | 161 | MC-SC |
| Val98 (O) | Lys56 (NZ) | 3.02 | 145 | MC-SC |

a) N, O, C are the donor-acceptor atoms; from general statistics this angle is 149° ± 15° for MC-MC hydrogen bonds and 129° ± 18° for SC-MC and SC-SC H-bonds;
b) MC (main chain) and SC (side chain) donor/acceptor atoms;
