# Supplementary material for: A silent H-bond can be mutationally activated for high-affinity interaction of BMP-2 and activin type IIB receptor
Source: BMC Struct Biol. 2007 Feb 12;7:6. doi: 10.1186/1472-6807-7-6 (PMC1802081; doi:10.1186/1472-6807-7-6)
Supplement: Additional File 5 — Binding of the type II receptor is dominated by hydrophobic interactions. Thermodynamic parameters of the interaction of ActR-IIBECD with the binary complex BMP-2:BMPR-IAECD were determined by isothermal calorimetry (ITC) [file 1472-6807-7-6-S5.pdf]

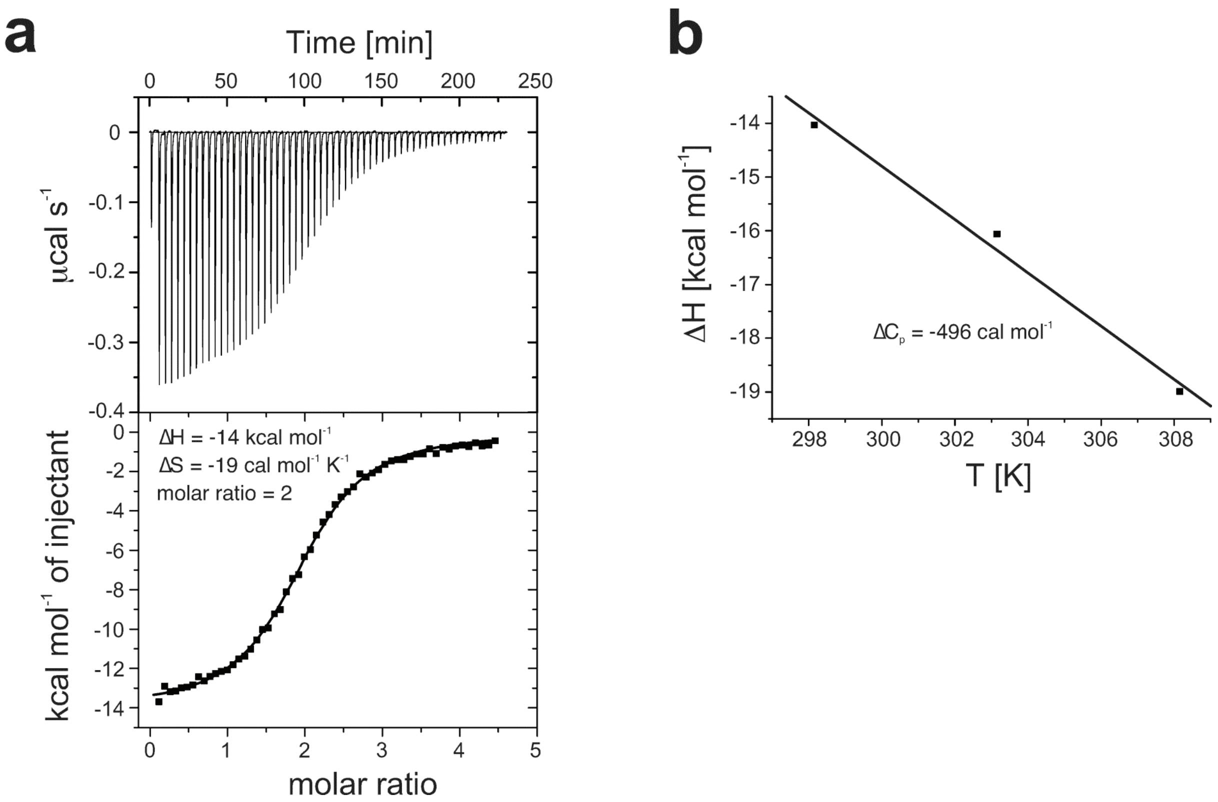

Binding of the type II receptor is dominated by hydrophobic interactions

Thermodynamic parameters for ternary complex formation were determined using a VP-ITC microcalorimeter (MicroCal Inc., Northampton, MA, USA) at 25°C, 30°C, and 35°C, respectively. All solutions used for titration experiments were degassed at 20°C for 10min. For the binding of ActR-IIB_{ECD} to the BMP-2:BMPR-IA_{ECD} complex, both proteins were dialyzed against the same batch of HBS_{500} buffer (10mM HEPES, 500mM NaCl, pH7.4). The syringe contained 150µM ActR-IIB_{ECD}, the concentration of the binary complex BMP-2:BMPR-IA_{ECD} in the measurement cell was 7.5µM. One preliminary injection (3µl) of ActR-IIB_{ECD} was followed by 56 injections of 5µl aliquots with an injection flow rate of 0.5µl s^{-1}. The stirring speed was set to 300rpm, the equilibration period between the injections was 240s. The results of the protein titration experiments were corrected by subtraction of the heats of dilution. Data analysis was preformed using the software MicroCal Origin (version 7.0). Data points were fitted using a one-site-binding model. (a) An isothermal titration experiment for the interaction of ActR-IIB_{ECD} with the binary BMP-2:BMPR-IA_{ECD} complex at 298K. The upper panel shows the consecutive injections of ActR-IIB_{ECD} to the binary complex of BMP-2 and BMPR-IA_{ECD} resulting in heat release. The lower panel shows the processed data. (b) The heat capacity change ΔC_p (the temperature dependency of the

enthalpy) was determined for the ActR-IIB$_{ECD}$ - BMP-2:BMPR-IA$_{ECD}$ interaction from a titration series at three different temperatures (T = 298K, 303K and 308K). The heat capacity, indicated in the diagram, can be deduced from the slope of a graph enthalpy (ΔH) versus measurement temperature.
