# Supplementary material for: A silent H-bond can be mutationally activated for high-affinity interaction of BMP-2 and activin type IIB receptor
Source: BMC Struct Biol. 2007 Feb 12;7:6. doi: 10.1186/1472-6807-7-6 (PMC1802081; doi:10.1186/1472-6807-7-6)
Supplement: Additional File 6 — MAD data for the ternary complex (1:1:1) BMP-2:BMPR-IAECD:ActR-IIBECD. Acquisition and processing statistics on a 3-wavelength MAD dataset used to solve the structure of the ternary complex (1:1:1) of wildtype BMP-2 [file 1472-6807-7-6-S6.pdf]

MAD data for the ternary complex (1:1:1) BMP-2:BMPR-IA$_{ECD}$:ActR-IIB$_{ECD}$

Crystal	BMP-2:BMPR-IA$_{ECD}$(F35M, L73M, L95M):ActR-IIB$_{ECD}$		
Space group	$P2_12_12_1$		
Cell constants	a = 64.36Å, b = 65.68Å, c = 113.97Å<br>$\alpha = \beta = \gamma = 90°$		
	Se-Met (λ1)	Se-Met (λ2)	Se-Met (λ3)
Wavelength	0.9799 Å	0.9796 Å	0.9079 Å
Resolution (Å)[a]	30.0 – 2.75 Å<br>(2.90 – 2.75 Å)	30.0 – 2.75 Å<br>(2.90 – 2.75 Å)	30.0 – 2.75 Å<br>(2.90 – 2.75 Å)
	λ1 (inflection)	λ2 (peak)	λ3 (remote high)
Number of measured reflections[b]	183773 (26598)	185933 (26610)	188670 (27520)
Number of unique reflections[b]	13130 (1874)	13134 (1872)	13137 (1879)
Completeness	99.9 (99.9)	99.9 (99.9)	99.9 (99.9)
Multiplicity	14.0 (14.2)	14.2 (14.2)	14.4 (14.9)
R_{sym} for all reflections[c]	6.1 (16.6) %	7.1 (18.7) %	6.7 (17.4) %
<Intensity/σ>	38.3 (14.8)	33.6 (13.2)	35.7 (14.8)
Phasing		7 out of 10 Se-sites identified	
R_{cullis} (a/c)[d]	0.469/0.401	0.586/0.590	-
R_{Kraut}[e]	0.025	0.022	0.039
Phasing power (a/c)[f]	3.07/3.30	2.26/2.31	1.50/1.72
Figure of merit after DM		0.75	

a) Numbers in parentheses indicate highest resolution shell

b) Cut-off for reflections $F > 0\sigma$

c) $R_{sym} = \sum_{hkl}|I_{hkl} - \langle I_{hkl}\rangle| / \sum_{hkl}\langle I_{hkl}\rangle$ where $\langle I_{hkl}\rangle$ is the mean intensity of symmetry related observations of a unique reflection

d) R_{cullis} = <phase-integrated lack of closure>/$\langle F_{PH} - F_P\rangle$
(a/c) denotes acentric and centric reflections

e) $R_{Kraut} = \sum |(|F_P+F_H|)-|F_{PH}|)|/\sum|F_P|$

f) Phasing power = $(\sqrt{|F_H^2|})/(\sqrt{|\text{lack-of-closure}^2|})$, (a/c) denotes acentric and centric reflections
